# Nanoscale Rheology: Dynamic Mechanical Analysis over a Broad and Continuous Frequency Range Using Photothermal Actuation Atomic Force Microscopy

**DOI:** 10.1021/acs.macromol.3c02052

**Published:** 2024-01-16

**Authors:** Alba R. Piacenti, Casey Adam, Nicholas Hawkins, Ryan Wagner, Jacob Seifert, Yukinori Taniguchi, Roger Proksch, Sonia Contera

**Affiliations:** † Clarendon Laboratory, Department of Physics, University of Oxford, OX1 3PU Oxford, U.K.; ‡ Department of Engineering Science, University of Oxford, OX1 3PJ Oxford, U.K.; § School of Mechanical Engineering, Purdue University, West Lafayette, Indiana 47907, United States; ∥ Asylum Research, Oxford Instruments KK, Tokyo 103-0006, Japan; ⊥ Asylum Research – An Oxford Instruments Company, Santa Barbara, California 93117, United States

## Abstract

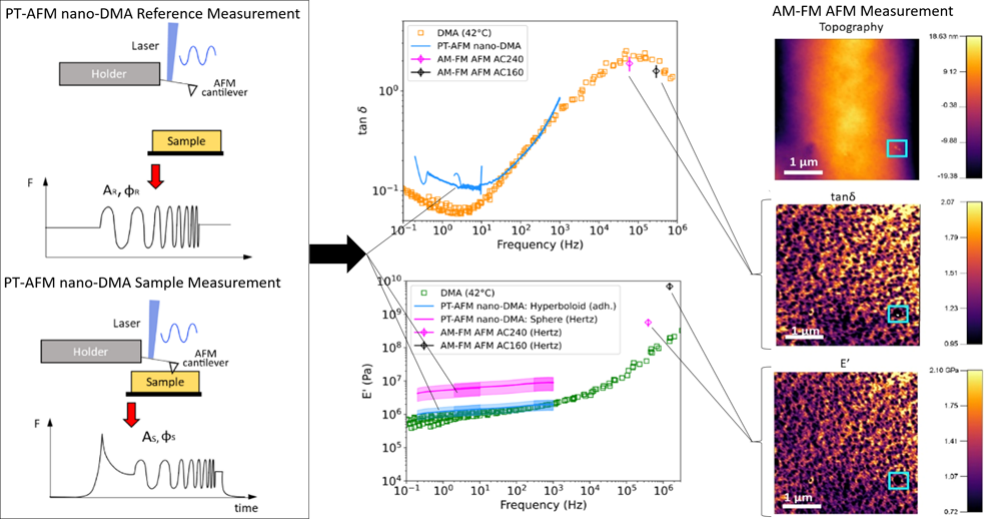

Polymeric materials are widely used in industries ranging from automotive to biomedical. Their mechanical properties play a crucial role in their application and function and arise from the nanoscale structures and interactions of their constitutive polymer molecules. Polymeric materials behave viscoelastically, i.e., their mechanical responses depend on the time scale of the measurements; quantifying these time-dependent rheological properties at the nanoscale is relevant to develop, for example, accurate models and simulations of those materials, which are needed for advanced industrial applications. In this paper, an atomic force microscopy (AFM) method based on the photothermal actuation of an AFM cantilever is developed to quantify the nanoscale loss tangent, storage modulus, and loss modulus of polymeric materials. The method is then validated on styrene–butadiene rubber (SBR), demonstrating the method’s ability to quantify nanoscale viscoelasticity over a continuous frequency range up to 5 orders of magnitude (0.2–20,200 Hz). Furthermore, this method is combined with AFM viscoelastic mapping obtained with amplitude modulation–frequency modulation (AM–FM) AFM, enabling the extension of viscoelastic quantification over an even broader frequency range and demonstrating that the novel technique synergizes with preexisting AFM techniques for quantitative measurement of viscoelastic properties. The method presented here introduces a way to characterize the viscoelasticity of polymeric materials and soft and biological matter in general at the nanoscale for any application.

## Introduction

Polymeric materials are widely used in many different types of applications and exhibit time- and frequency-dependent mechanical behavior known as viscoelasticity.^1^ The viscoelastic properties of polymeric materials are crucial to their function and application and arise from the structure and interactions of polymers within the material.^1,2^ Quantifying material viscoelasticity is therefore essential in determining the material’s application and in providing insight into the material’s structure.^1,2^ Typically, viscoelasticity is quantified at the macroscale using techniques such as dynamic mechanical analysis (DMA) or rheometry.^1,3^ However, it is also useful (e.g., to construct or validate predictive models of polymeric behavior), though more technically demanding, to quantify viscoelasticity at the nanoscale since this is the length scale at which polymers interact within the material. In both macro- and nano-DMA or rheology, an axial or torsional stimulus is applied to the sample. For macroscale measurements, a large, typically mm, stimulus is applied to the sample.^1,3^ For nano-DMA, the stimulus is applied to a localized position on the sample, typically nm or μm in size, by a nanoscale or microscale size probe; some methods also stimulate the whole sample and measure its response locally, using the probe.^4,5^

Among techniques to quantify the nanoscale viscoelasticity of polymeric materials, atomic force microscopy (AFM) is one of the most versatile techniques. In AFM, it is possible to apply a wide range of forces, from pN to μN, to a sample and probe samples at different length scales, from nm to hundreds of μm, depending on the stiffness, tip size, and shape of the AFM cantilever.^6,7^ Moreover, localized AFM measurements can be combined to create quantitative maps of a sample’s mechanical properties with high spatial resolution.^7^ Furthermore, AFM can be used in liquid and at different temperatures,^6^ allowing inert materials and biological (even living) samples to be measured in conditions similar to those of their application. Lastly, in general, the AFM requires no external fields that might interfere with the natural behavior of the studied material.

Several AFM techniques, including contact resonance (CR) and multifrequency AFM, have been used to map the viscoelastic properties of samples at frequencies corresponding to the AFM cantilever’s harmonics or eigenmodes.^8−16^ However, measuring properties over a wide frequency range is preferred because sample viscoelasticity is frequency-dependent. The wider the measured frequency range, the more is known about a material’s viscoelastic behavior and its relation to the internal molecular structure. In recent years, off-resonance AFM nano/microrheology has been developed to study the viscoelastic properties of many different materials, including rubbers,^17−22^ cells,^23−27^ single cell nuclei,^28^ cartilage,^29−33^ and polymer gels.^23,34^ However, there are limitations shared by existing AFM nano/microrheology techniques. The first limitation is that the frequencies over which properties can be measured are limited by reliance on piezoelectric (PE) actuators to excite the cantilever. PE actuators can introduce spurious peaks in the cantilever’s oscillatory spectrum, especially in liquid,^35−37^ thereby causing noise in rheological measurements and rendering experiments unreliable or difficult to analyze (especially on biological samples). So far, different solutions have been used to overcome the limited frequency range of PE-actuated systems, including an adaptation of high-frequency piezo actuators,^18−20,26^ compensation for PE resonances,^27^ application of the time–temperature superposition (TTS) principle,^17,18,21,22^ or using direct cantilever excitation via magnetic actuation.^34^ Nevertheless, the spurious spectrum emerging from unwanted resonances still limits PE methods, and TTS or magnetic actuation might alter sample behavior by compromising the material via temperature change (e.g., leading to DNA/biomolecular denaturation), application of magnetic fields (e.g., magneto-active materials), and biotoxicity of magnetic coatings of cantilevers.

Photothermal (PT) actuation is another way of directly exciting AFM cantilevers,^38,39^ and is already used in commercial instruments to quantify the mechanical properties of materials both on-resonance^15,40^ and off-resonance^41,42^ and is particularly useful for biological samples in liquid environments.^16^ In this paper, we develop a nano/microscale rheology AFM technique using PT cantilever actuation and show that this method can accurately measure the viscoelastic properties of a sample over a continuous and wide frequency range of 5 orders in magnitude.

## Results and Discussion

### PT-AFM Nano-DMA

**Figure 1:**
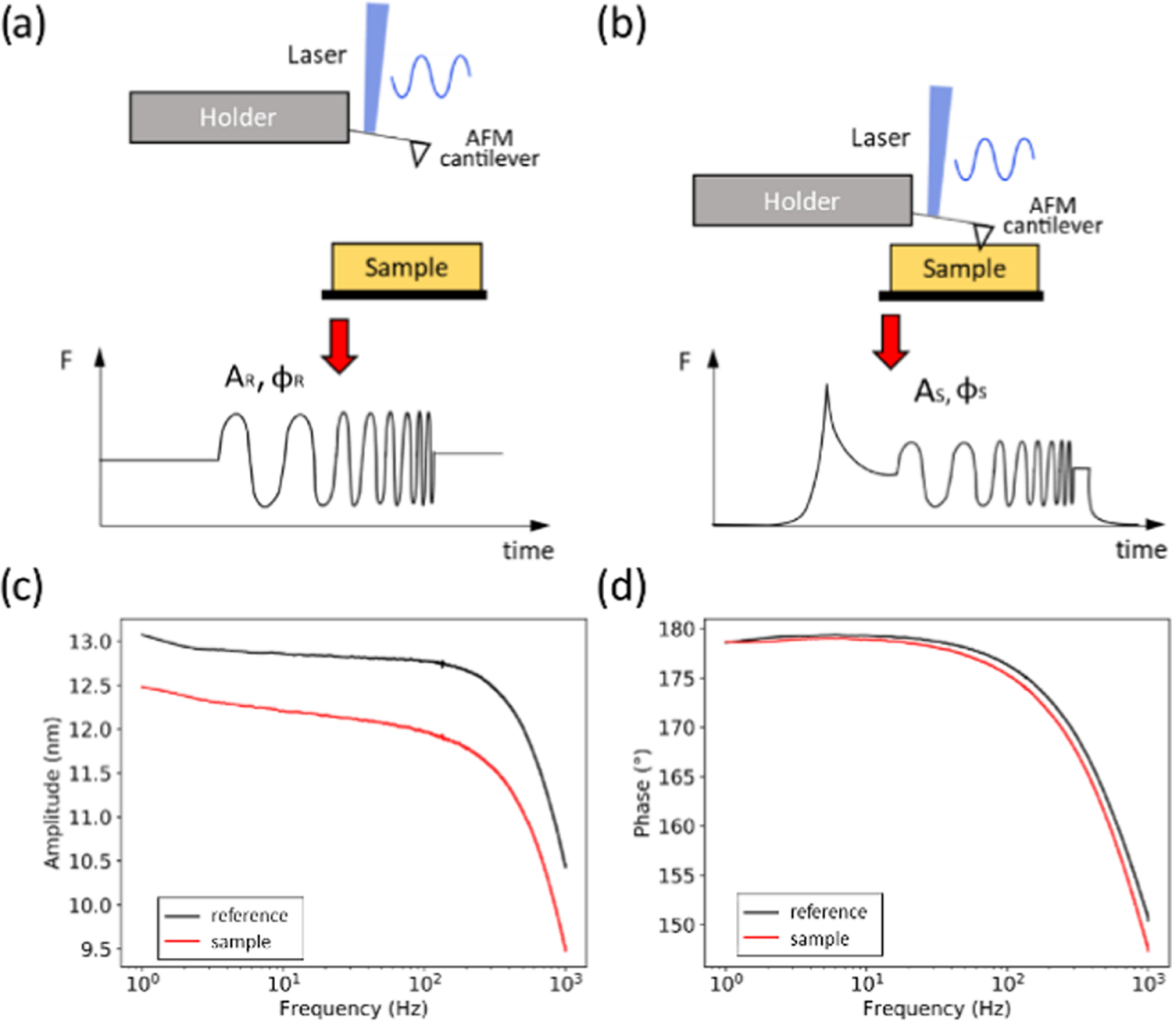
Principle of PT-AFM nano-DMA (photothermal atomic force microscopy nanodynamic mechanical analysis). A reference measurement, where the cantilever is excited by a chirped oscillation while not in contact with the sample (a) is compared to a sample measurement, where the cantilever is excited by the same chirped oscillation while in contact with the sample (b). Schematics of the force (*F*) experienced by the cantilever are shown at the bottom of panels (a) and (b). Cantilever amplitude (*A*) and phase (φ) vary between the reference (R) and sample (S) measurements. The representative amplitude and phase changes between the sample (a styrene–butadiene rubber (SBR), red) and reference (black) measurements are shown in panels (c) and (d), respectively. Sample viscoelasticity is calculated by comparing *A*_R_ and φ_R_ with *A*_S_ and φ_S_.

The principle of our PT-AFM nano-DMA technique is shown in Figure 1. PT cantilever excitation is achieved by modulating the power of an excitation laser (EL). The PT-EL is focused on the back of the cantilever and drives cantilever motion by PT excitation.^38,39^ To obtain a continuous wide spectrum of frequencies, the PT-EL is modulated with exponentially chirped oscillations at frequencies well below the cantilever’s resonance.^41^ Details on why exponential chirps were used, as well as an analysis of the effects of PT-EL power and positioning, are provided in the Supporting Information SI3 and SI4. Comparing chirped cantilever oscillations above (Figure 1a) and during indentation (Figure 1b) of the sample allows quantification of the sample viscoelasticity. The measurement above the sample while the cantilever is out of contact acts as a reference measurement. The measurement performed while indenting, and being in contact with the sample, is the sample measurement. A detailed analysis of factors that can influence cantilever motion (e.g., PT-EL position, PT-EL displacements during cantilever motion, and the extent by which tip/sample separation affects the reference signal) and sample response (e.g., nonlinear viscoelastic behavior due to high strains caused by large oscillations or indentations), and hence influence PT-AFM nano-DMA measurements, is provided in the Supporting Information SI4, and shows that PT-AFM nano-DMA is robust to most of these factors, and reliably quantifies sample viscoelasticity.

Figure 1c,d shows the representative amplitude and phase signals of chirped AFM cantilever (an AC160TSA) oscillations during a reference measurement and during the indentation of a styrene–butadiene rubber (SBR) sample. For both measurements, the amplitude and phase decrease with increasing frequency, which is typical of PT measurements.^43^ The differences between the amplitude and phase between the sample and reference measurements are used to calculate the sample viscoelasticity. As derived by Nalam et al.,^34^ the real (*k*′) and imaginary (*k*″) components of the dynamic stiffness of a sample (k* = *k*′ + i*k*″) probed with sinusoidally directly actuated cantilevers can be calculated from amplitude (*A*) and phase (φ) as follows:
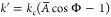
1
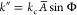
2where *A̅* = *A*_R_/*A*_S_ and Φ = φ_R_ – φ_S_, with A and φ being the amplitude and phase of the oscillations measured out of contact (reference measurement, subscript R) and in contact (sample measurement, subscript S) with the sample.

To directly apply eqs 1 and 2 to PT-AFM nano-DMA, the optical lever calibration of both *A*_R_ and *A*_S_ must be identical. This calibration depends on the shape in which the cantilever vibrates, which generally changes with the photothermal laser spot position, drive frequency, and cantilever boundary conditions^41,44^ (these factors are analyzed further in Supporting Information SI4). Without correcting for these shape changes, measurements at different ratios of |k*|:*k*_c_ are not directly comparable. However, if *k*_c_ ≫ \k*|, the change in the cantilever vibration shape due to changes in sample stiffness is small.^41^ Selecting |k*|/*k*_c_ ratios that are sensitive to amplitude changes but insensitive to vibration shape changes and comparing *A*_R_ and *A*_S_ at matching frequencies are, therefore, essential for PT-AFM nano-DMA to provide accurate results. Examples that illustrate what happens when these conditions are violated are demonstrated in Supporting Information SI4.

The loss tangent, tan δ, of a sample can be calculated as the ratio of the imaginary and the real components of k* as follows:^19^
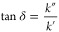
3Importantly, contrary to *k*′ and *k*″, tan δ does not depend on the geometry of the system,^3^ in this case, the tip/sample contact. Calculation of the measurement uncertainty in *k*′ and *k*″ can be found in the Supporting Information SI5.

**Figure 2:**
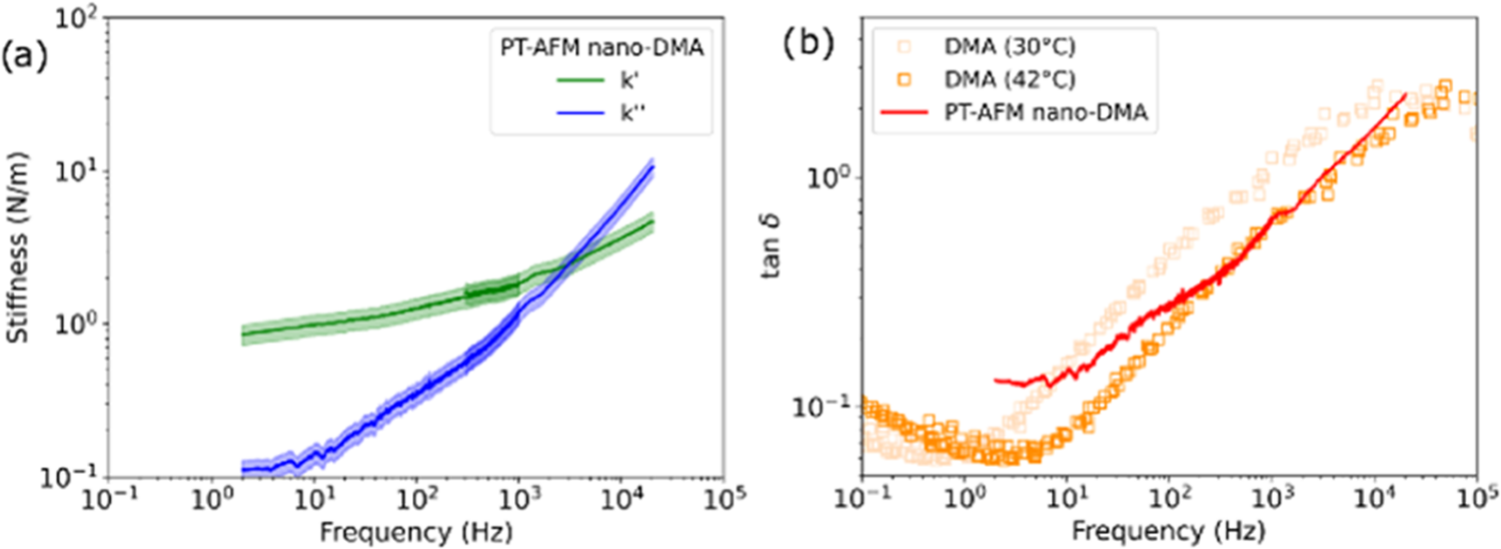
SBR *k*′, *k*″, and tan δ measured using PT-AFM nano-DMA. The storage stiffness (*k*′, green line) and loss stiffness (*k*″, blue line) (a). Comparisons between the loss tangent (tan δ) measured with PT-AFM nano-DMA (red line) and macroscopic DMA (unfilled squares) master curves with reference temperatures of 42 °C (dark orange) and 30 °C (peach) (b). Shading represents the experimental error, calculated as detailed in Supporting Information SI5, of PT-AFM nano-DMA measurements.

Figure 2a shows SBR *k*′ and *k*″ calculated using eqs 1 and 2. Both values increase with increasing frequency, with *k*″ becoming larger than *k*′ above 2 kHz; tan δ was then calculated using eq 3 and compared with control SBR measurements obtained via macroscale DMA (Figure 2b). PT-AFM nano-DMA measurements are comparable with macroscopic DMA data at 42 °C rather than at 30 °C (ambient room temperature), suggesting a local increase in sample temperature during PT-AFM nano-DMA measurements. This local heating is likely due to the PT-EL, as further described in Supporting Information SI4. At frequencies less than 1 Hz, tan δ measured with PT-AFM nano-DMA deviated slightly from macroscale DMA measurements. However, small deviations between local and macroscopic rheological measurements have been previously reported^19,21,34^ in some cases attributed to nonlinear viscoelastic effects.^21^ However, as demonstrated in Supporting Information SI4, nonlinear effects are avoided in our SBR measurements. More likely, the deviation between local and macroscopic DMA measurements of the SBR is due to surface effects that come into play at smaller length scales.^34^ Regardless of these differences, macroscopic measurements could potentially miss features relevant at smaller length scales by averaging over a larger scale. Furthermore, the bulk oscillation of the whole sample is also likely to affect the local mechanical properties in a different way than nanoscale oscillations confined to the place of the measurement. Small deviations at high frequencies (approximately 10–20 kHz) could similarly be due to surface effects, although |k*| approaching the value of *k*_c_ might also be affecting the measurements (see Supporting Information SI4).

**Figure 3:**
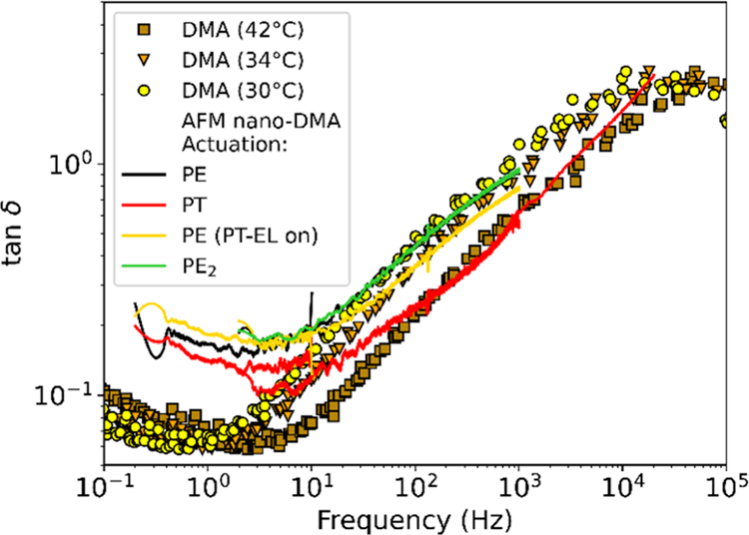
Evaluation of PT-AFM nano-DMA against PE-AFM nano-DMA and macroscale DMA measurements of the SBR. AFM nano-DMA measurements were obtained with PE (black line) and PT (red line) excitation. PE measurements were performed first. Then, PT measurements were performed. Next, PE measurements were performed while the PT-EL was still focused on, but not exciting, the cantilever (gold line). Finally, PE measurements were repeated after deactivation of the PT-EL (green line). Macroscopic DMA (filled markers) master curves with reference temperatures of 42 °C (brown squares), 34 °C (orange triangles), and 30 °C (yellow circles) are also shown as controls for nano-DMA measurements.

To evaluate how PT measurements compare with PE measurements, AFM nano-DMA measurements of the SBR were obtained using PE actuation via sample modulation and compared to PT-AFM nano-DMA measurements. Additionally, PE-AFM nano-DMA measurements were used to provide insight into the local temperature increase caused by the PT-EL. Cantilever excitation was chosen to keep similar reference amplitudes for PE and PT measurements (see Supporting Information SI1). PE and PT measurements were performed on the same spot in the SBR sample. PE measurements were performed first (i), followed by PT measurements (ii). Then, PE measurements were performed, keeping the PT-EL focused on the cantilever with only DC power (iii), that is, with the PT-EL but without any applied PT-EL oscillation. Finally, the PT-EL was deactivated, and PE measurements were performed a second time on a different spot on the sample (iv). The relationships obtained by Igarashi et al.^19^ were used to calculate *k*′ and *k*″ from PE measurements. As shown in Supporting Information SI1, data obtained with PE actuation could not be measured above 1 kHz due to the presence of spurious resonances arising from the PE actuator-AFM coupled system;^35−37^ tan δ values measured by (i–iv) are shown in Figure 3. Unlike PT data (ii), PE data (i) agree well with macroscopic DMA at 30 °C. PE data obtained when the PT-EL was focused on the cantilever but not used to excite the cantilever (iii) matches DMA data at the intermediate temperature of 34 °C. After switching off the PT-EL, another PE measurement was performed (iv), and the data once again overlapped well with DMA at 30 °C. These observations from (i)–(iv) indicate that the PT-EL causes local sample heating. Nevertheless, it would be worth investigating this effect further, either using thermocouples in proximity to the sample/cantilever or using AFM cantilevers with integrated thermometers.^45,46^ It is important to note that if PT-EL power is known, it may be possible to find a relation between the local temperature increase and PT-EL power. However, this calculation is not straightforward because a variety of variables (including sample properties, the properties of the sample’s moisture layer, proximity to the sample, and cantilever properties) can influence this relation. Additionally, different AFMs might have different PT-EL powers.^47^ Therefore, for a more accurate idea of PT-EL power changes, we use the percent of maximum PT-EL power by applying different voltages to the PT-EL photodiode, as described in Supporting Information SI4. Relating PT-EL power to sample heating is, therefore, beyond the scope of this work. However, previous analysis of cantilever temperature during PT excitation in liquid shows that cantilever temperature increases by several degrees.^48^ Dry, these effects may be even more pronounced.

### Dynamic Modulus

Typically, viscoelastic materials are not described in terms of *k*′ and *k*″, but instead by their dynamic modulus (E* = *E*′ + i*E*″).^1^ The real and imaginary components of E* are called the storage (*E*′) and the loss (*E*″) moduli and, respectively, represent the elastically stored energy density and the energy density dissipated during sample deformation.^1,3^E* can be calculated from k* by applying a contact model to describe the indenter–sample system. The presence of adhesive forces can complicate the contact model equations.^19^ For dynamic experiments on adhesive viscoelastic materials, as long as the oscillation frequency is high enough, viscoelastic effects cause indenters of different shapes to behave like a flat cylindrical punch (i.e., constant contact radius) during the oscillations.^49,50^ For these cases, the relation between E* and k* can be written as follows (this equation also applies to other indenter shapes, such as spherical):^49,50^
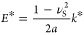
4where *a* is the contact radius between the indenter (I) and the sample (S) during the oscillations and ν_S_ is the Poisson’s ratio of the sample. In this paper, ν_S_ = 0.5 was assumed, which is typical of SBRs.^19−22^ From eq 4, it can also be seen that, for a relationship where E* and k* are directly proportional, eq 3 results in tan δ = *k*″/*k*′ = *E*″/*E*′, which is the usual definition of tan δ.^1−3^

To calculate *a*, it is necessary to determine the correct contact model to describe the system. For soft and adhesive rubbery materials like the SBR, it is appropriate to use contact models such as the Johnson–Kendall–Robertson (JKR) model,^19^ originally introduced for deformation of spherical bodies.^51^ In general, the following relationships can be used to describe the relationship between the force *F* exerted by a rigid indenter and the deformation *d* of an elastic half space^52^
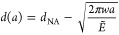
5
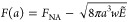
6where *d*_NA_ and *F*_NA_ are the deformation and the force calculated in the nonadhesive case, respectively, *w* is the energy of adhesion per unit contact area, and *Ẽ* is the reduced Young’s modulus defined as^6^, if *E*_I_ ≫ *E*_S_ (as usual in AFM experiments).

**Figure 4:**
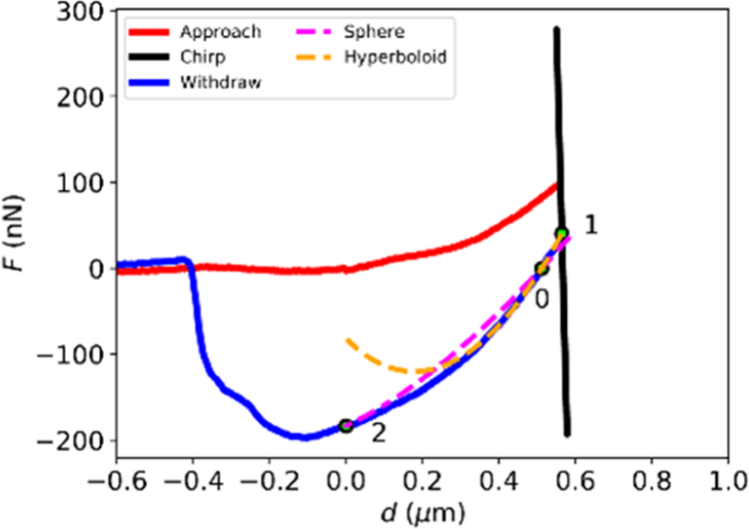
Typical force (*F*) vs. indentation (*d*) curve of the SBR from a PT-AFM nano-DMA experiment using an AC160 cantilever and a chirp frequency range of 0.1–10 Hz. The approach (red line), chirped oscillations (black line), and withdrawal (blue line) curves are shown. Point “0” corresponds to the point of zero load, “1” to the average of the oscillatory force (i.e., the point around which dynamic oscillations occur), and “2” to the point of zero indentation. Dashed lines represent contact model fits, evaluated by calculating *w* and *Ẽ* for a spherical JKR contact geometry (pink line) and a hyperboloid (orange line) contact geometry.

For indenters with hyperboloid shapes, Sun et al.^53^ proposed a model that can be simplified to the following (details in Supporting Information SI2)
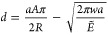
7
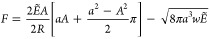
8where *A* = *R* cot α, with α being the indenter semivertical angle and *R* the tip radius. For AC160 and AC240 cantilevers, *R* was assumed to be the nominal cantilever radius (*R* = 7 nm) and α was taken as half of the tip’s nominal back angle (α = 17.5°). Note that while the tips used in our experiments have a tetrahedral geometry, as described by the manufacturer, it is, however, impossible to measure the actual geometry at the nanoscale contact. The hyperboloid model has been shown to be a good approximation (eqs 7 and 8) of the contact geometry of our system, as demonstrated by the fitting to the experimental data presented in Figure 4, and in good agreement with macroscopic DMA measurements.

To calculate *w* and *Ẽ* necessary to obtain *a*, and therefore *E*′ and *E*″, the “two-points method,”^53^ which relies on particular points in force indentation withdrawal curves, can be used.^19^ After the contact points of the curves are identified, as described in the Experimental Methods section, useful points can be identified in the curves. In Figure 4, the relevant points are marked in a typical force indentation curve obtained for a PT-AFM nano-DMA experiment on the SBR: the point of zero load “0,” the point around which dynamic oscillations occur “1,”^19^ and the point of zero deformation “2.” Figure 4 also compares the measured withdraw curve to the curves for the spherical JKR (eqs 5 and 6) and hyperboloid (eqs 7 and 8) contact models evaluated using the quantities calculated with the “two-points method”^53^ (see Supporting Information SI2). The hyperboloid model, calculated using point “0” and point “1” as described in Supporting Information SI2, agrees well with the experimental curve for larger indentations. This is expected since the hyperboloid model used in this paper (detailed in Supporting Information SI2) assumes that *a* ≫ *A*, which is most likely to apply to large indentations. Therefore, for large indentations, the hyperboloid model is better suited than the JKR model because the JKR model is an extension of the Hertz model, which assumes deformations much smaller than the tip radius.^51,54,55^

**Figure 5:**
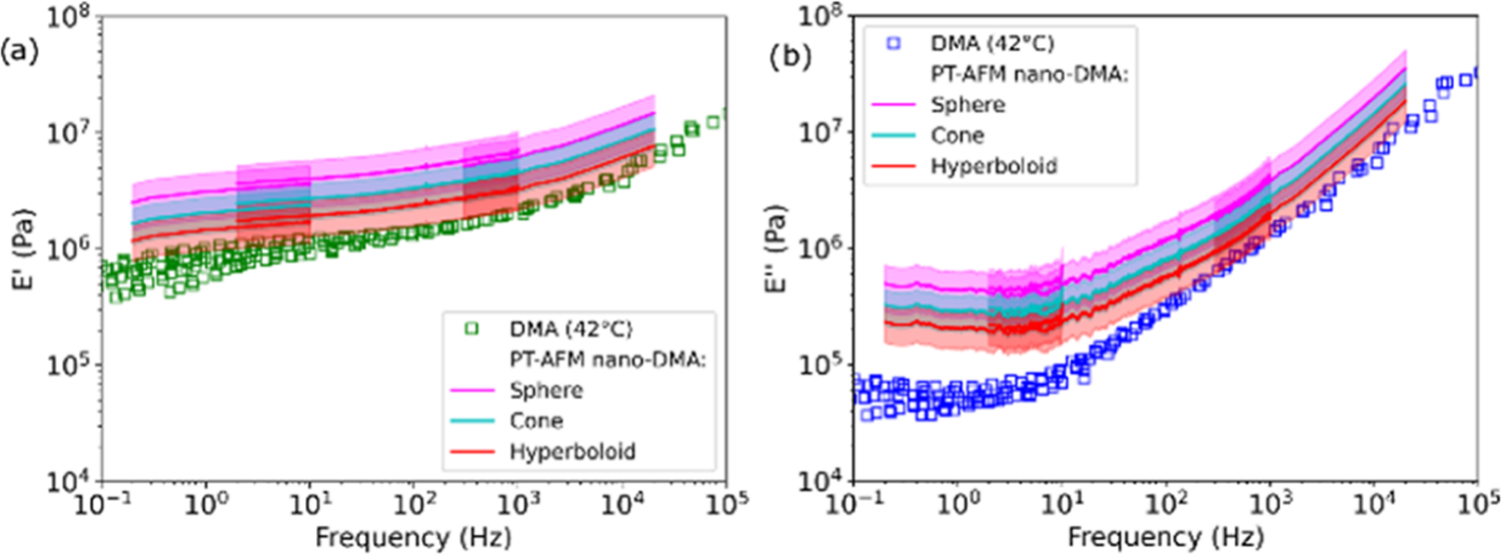
SBR storage (*E*′) and loss (*E*″) moduli measured by PT-AFM nano-DMA (continuous lines) using different contact models. (a) *E*′, and (b) *E*″ were calculated from *k*′ and *k*″ via eq 4 for adhesive contacts with spherical (pink line), conical (cyan line), and hyperboloid (red line) indenters (see Supporting Information SI2). Measurement errors (see Supporting Information SI6) are shown as shades. Unfilled squares represent the macroscopic DMA control measurement at 42 °C.

For PT-AFM nano-DMA, calculating *E*′ and *E*″ (eq 4) requires knowledge of *a* in point “1” (*a*_1_). The value of *a*_1_ was calculated for a spherical, conical, and hyperboloid indenter once *w* and *Ẽ* were obtained for each geometry, as detailed in Supporting Information SI2. The procedure to calculate the uncertainty in *E*′ and *E*″ is described in Supporting Information SI6. The resulting SBR *E*′ and *E*″ are shown as lines in Figure 5a,b and compared to the macroscopic DMA control (unfilled squares). *E*′ and *E*″ were overestimated by PT-AFM nano-DMA when *a*_1_ was calculated via the spherical and conical indenter models, most likely because the contact geometry is not well described by these two models. *E*′ and *E*″ calculated using *a*_1_ calculated with the hyperboloid contact model are in good agreement with the macroscopic DMA data. Slight deviations between moduli measured with PT-AFM nano-DMA and macroscopic DMA at low frequencies can be explained as proposed above for tan δ measurements.

It is important to note that other contact models could also be employed to describe the SBR tip/sample contact. For example, the indentation curve shown in Figure 4 potentially exhibits characteristics of plastic deformation.^6^ However, since multiple *F* vs. *d* curves performed in succession exhibited the same shape (shown in Figure 4), which would not be the case for plastic deformation, since the hyperboloid with adhesion fits the *F* vs. *d* curves well (Figure 4), and since the resulting SBR *E*′ and *E*″ match control DMA measurements (Figure 5), it is reasonable to conclude that plastic deformation did not significantly impact PT-AFM nano-DMA measurements of this sample.

### AM–FM AFM Imaging

**Figure 6:**
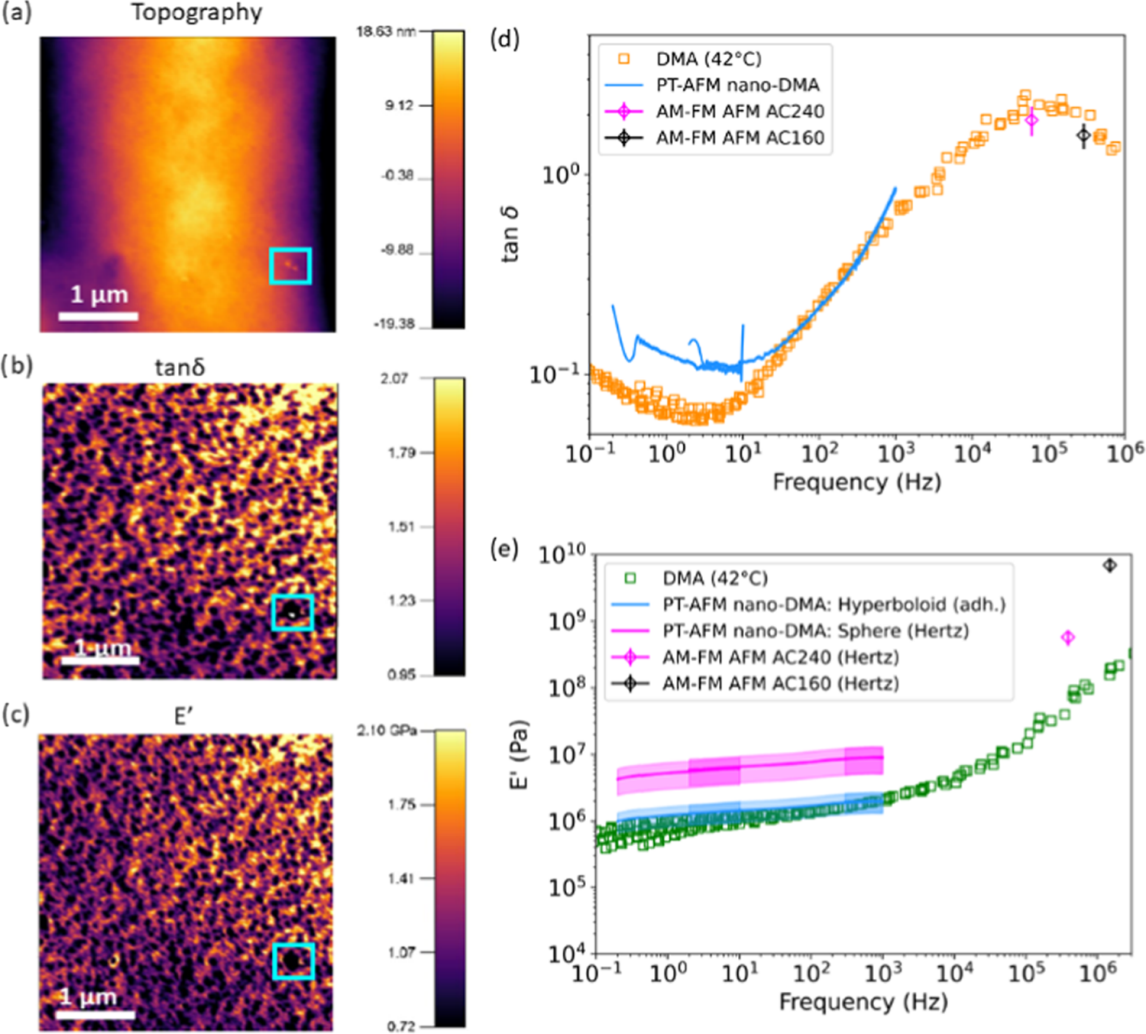
Synergy of PT-AFM nano-DMA and AM–FM AFM measurements. Representative AM–FM AFM maps of SBR topography, loss tangent (tan δ), and storage modulus (*E*′) ((a)–(c), respectively). Blue squares in panels (a–c) indicate how SBR surface features affect tan δ and *E*′ values measured by AM–FM AFM. These images were collected with an AC240TSA cantilever but are representative of all AM–FM AFM images of the SBR surface regardless of the cantilever. Comparisons of tan δ and *E*′ values measured by different techniques, are respectively shown in (d) and (e). Control macroscopic DMA data (42 °C) are displayed as unfilled squares. AM–FM AFM measurements collected with AC240 (pink) and AC160 (black) cantilevers are shown as points with error bars representing the mean ± standard deviation. PT-AFM nano-DMA data collected with an AC240TSA are displayed as curves with shading (mean ± measurement error). PT-AFM nano-DMA collected with an AC160TSA is displayed in the previous figures. In panel (e), PT-AFM nano-DMA *E*′ is calculated for both a nonadhesive contact with a spherical indenter (Hertz model, pink line) and an adhesive contact with a hyperboloid indenter (cyan line).

In order to evaluate how PT-AFM nano-DMA measurements compare to other AFM techniques that are used to measure viscoelastic properties of soft materials, the SBR sample was also measured by using amplitude modulation–frequency modulation (AM–FM) AFM. AM–FM AFM is an on-resonance technique that measures sample viscoelasticity by simultaneously driving the cantilever at two of its eigenmodes, typically the first and second.^14^ AM–FM AFM allows quantitative mapping of sample topography, tan δ, and *E*′.^13−15^ The first, lower-frequency mode is subject to amplitude modulation and measures sample topography and tan δ.^56,57^ The second, higher-frequency mode is subject to frequency modulation and, combined with parameters from the first mode, measures sample *E*′ by applying the Hertz contact model.^14,15^ While both modes contribute to the calculation of *E*′, the second mode contributes the most.^14^ Hence AM–FM AFM measures sample tan δ at the lower frequency and *E*′ at the higher frequency.^14,15,58^Figure 6a–c shows representative AM–FM AFM maps of the SBR. The images in Figure 6a–c were obtained using an Olympus AC240 cantilever (nominal *k*_c,1_ ∼ 2 N/m, *k*_c,2_ ∼ 50 N/m, first resonance frequency ∼70 kHz, second resonance frequency ∼400 kHz), but similar results were obtained using an Olympus AC160 cantilever (nominal *k*_c,1_ ∼ 26 N/m, *k*_c,2_ ∼ 364 N/m, first resonance frequency ∼300 kHz, second resonance frequency ∼1.5 MHz). Spatial variation was present in the SBR topography, tan δ, and *E*′ throughout the SBR surface, as indicated by the color schemes in Figure 6a–c. Mean AM–FM AFM values, shown in Figure 6d,e, were calculated by averaging all pixels from multiple AM–FM AFM images acquired using AC240 (25 images total) and AC160 (13 images total) cantilevers. These mean AM–FM AFM values were then compared with PT-AFM nano-DMA performed with an AC240 cantilever and macroscale DMA control measurements. In Figure 6e, PT-AFM nano-DMA E′ was calculated via eq 4 for a nonadhesive contact with a spherical indenter (Hertz contact model,^54,55^ pink line) in order to apply the same contact model as AM–FM AFM measurements, as well as an adhesive contact with a hyperboloid indenter (cyan line, see Supporting Information SI2).

AM–FM AFM tan δ measurements with both AC240 and AC160 cantilevers agreed well with control macroscale DMA measurements (Figure 6b,d). However, AM–FM AFM *E*′ measurements with both cantilevers deviated from control DMA measurements (Figure 6c,e). The fact that tan δ is independent of the contact geometry and did not deviate from the DMA control, while *E*′ relies on a contact model and deviates from the control, suggests that the discrepancy between AM–FM AFM and macroscale DMA E′ is due to application of an inaccurate contact model to calculate AM–FM AFM *E*′. Supporting this conclusion, PT-AFM nano-DMA also overestimates SBR E′ compared to DMA control measurements when the Hertz model is used. PT-AFM nano-DMA E′ calculated using the adhesive hyperboloid model agrees well with macroscale DMA data, as was also the case with AC160 in Figure 5a. Therefore, an adhesive hyperboloid contact best describes the AC160/AC240 tip-SBR interaction, and the Hertz contact model used to calculate AM–FM AFM *E*′^14,15^ does not describe the tip/SBR interaction well. The discrepancy between AM–FM AFM and macroscale DMA *E*′ is due to the application of an oversimplifying contact model for AM–FM AFM calculations.

Figure 6 also shows that AM–FM AFM tan δ and *E*′ varied more with sample topography than PT-AFM nano-DMA, as indicated by the blue squares in Figure 6a–c. AM–FM AFM’s sensitivity to SBR topography is likely due to the fact that AM–FM AFM indentation depths are much smaller, a few nm, than AFM nano-DMA indentations (hundreds of nm, as shown in Figure 4), and therefore exhibit greater sensitivity to local properties and surface forces.^15,57^ Accounting for AM–FM AFM’s application of the Hertz contact model when measuring *E*′, and in spite of AM–FM AFM’s heightened sensitivity to surface effects, average AM–FM AFM tan δ and *E*′ values agreed with control DMA values and aligned with PT-AFM nano-DMA curves. Therefore, employing both AM–FM AFM and PT-AFM nano-DMA together enhances the amount of information obtained about the sample by measuring sample viscoelasticity over a frequency range larger than that of either technique alone while also acquiring information about sample topography. If sample topography is varied, performing AM–FM AFM before PT-AFM nano-DMA could also inform where to target PT-AFM nano-DMA measurements on the sample surface. Together, these results demonstrate that PT-AFM nano-DMA measurements synergize well with other AFM techniques that measure the sample viscoelasticity.

## Conclusions

In summary, we have shown that the PT actuation of AFM cantilevers can be employed to perform nano-DMA experiments. Performing PT actuation with chirp signals provided a way to achieve viscoelastic quantification over a continuous and wide 5 orders of magnitude frequency range. Measuring sample viscoelasticity over such a broad frequency range in a continuous fashion is advantageous because such a capability abolishes the need to construct master curves by taking advantage of the TTS principle to shift data measured from a range of temperatures to a reference temperature within that range, as is currently the case with macro-DMA measurements. Avoiding the creation of master curves while still measuring sample viscoelasticity over a broad and continuous frequency range increases the ease of measuring the viscoelastic properties of samples that are sensitive to temperature changes or to being clamped and exposed to large deformations within a DMA apparatus. Additionally, for samples that are less sensitive to temperature, it may be possible to further expand the frequency range of PT-AFM nano-DMA measurements by performing measurements at multiple different temperatures (imposed by a temperature-controlled stage) and constructing master curves for the sample. A comparison of PT-AFM nano-DMA with macroscopic DMA data and PE-AFM nano-DMA suggests that the PT laser causes a local increase in the sample temperature. Nevertheless, PT actuation does not involve magnetic fields that might potentially alter the sample and avoids the presence of spurious peaks in the cantilever’s spectrum typical of PE excitation. Therefore, despite the local temperature increase, the elimination of disrupting stimuli (magnetic fields) and spurious resonances render PT-AFM nano-DMA more versatile for nano-DMA measurements. We note that although the measurement sensitivity is expected to increase when *k*_c_ approaches |k*|^41,44^, the correct application of PT-AFM nano-DMA requires that *k*_c_ > |k*|. PT-AFM nano-DMA measurements are robust and synergize well with measurements collected via other AFM techniques such as AM–FM AFM. The combination of PT-AFM nano-DMA and AM–FM AFM enhances the amount of information obtained about the sample by measuring sample viscoelasticity over a larger frequency range than either technique alone while also acquiring information about sample topography. Additionally, PT-AFM nano-DMA could be employed in force mapping to enhance the quantification of sample mechanics at each point in the force map. To conclude, our novel PT-AFM nano-DMA technique serves as a useful tool to measure the nanoscale viscoelasticity of polymeric materials over a broad and continuous frequency range.

## Experimental Methods

### Sample

Styrene–butadiene rubber (SBR) was chosen as the test material for PT-AFM nano-DMA because SBR is already well characterized by AFM experiments.^17,19−22^ The SBR used was a random copolymer 36% styrene by weight, 57% 1,2-butadiene units in the butadiene fraction, and 43% 1,4-butadiene units in the butadiene fraction. The measured glass transition temperature was −13 °C, determined by differential scanning calorimetry (DSC Q1000, TA Instruments). Samples were cut to size (see experiments below), stored at −5 °C, and allowed to equilibrate at room temperature prior to measurements.

### Macroscale DMA Control

Macroscale DMA experiments were performed on SBR samples in air in order to serve as a control for nano-DMA measurements. A DMA Q800 (TA Instruments) in tension clamp configuration was used to perform DMA on an SBR sample 11.95 × 4.70 × 4.70 × 1.30 mm^4^ (*H*) in size. The sample was cooled to −50 °C, stabilized for 20 min, and then clamped. Frequency sweeps were performed at 0.1, 0.3, 1, 3, and 10 Hz in 2 °C steps from −50 to 70 °C. Before measurements at each step, the sample was allowed to equilibrate for 5 min. The strain was maintained at 0.1% to ensure that DMA was performed in the SBR’s linear viscoelastic regime. A 0.01 N preload force was used. The force track was set to 107%. TA Instruments Rheology Advantage Data Analysis Software (TA Instruments) was used to calculate the shift factors from the tan δ curve in order to generate DMA master curves. The master curves for *E*′, *E*″, and tan δ were calculated for a reference temperature *T*_0_ = 40 °C. For the TTS principle, the calculated shift factors can be used to shift the master curves for different temperatures.^1,3^

### AFM

AFM was performed on small SBR samples, roughly 2 mm (*L*) × 2 mm (*W*) × 3 mm (*H*) in size. All AFM experiments were performed in air at room temperature with a Cypher ES AFM (Oxford Instruments Asylum Research). AC160TSA-R3 (Olympus, nominal spring constant 26 N m^–1^, resonance frequency 300 kHz, tip radius 7 nm, tetrahedral tip shape), AC240TSA-R3 (Olympus, nominal spring constant 2 N m^–1^, resonance frequency 70 kHz, tip radius 7 nm tetrahedral tip shape), and biosphere-NT_B2000_v0010 (Nanotools, nominal spring constant 40 N m^–1^, resonance frequency 330 kHz, tip radius 2 μm, spherical tip shape, used in the Supporting Information SI4) cantilevers were used. Cantilever calibration was performed by indenting a hard substrate and using the thermal noise method.^59^ Data acquisition and cantilever calibration were performed using Oxford Instruments Asylum Research V16 software based on Igor Pro.

For AFM nano-DMA, sinusoidal excitations were applied to the cantilever. Sinusoidal cantilever excitation was achieved using exponential (also called logarithmic) chirped signals to provide mechanical measurements over a continuous and wide frequency range (Supporting Information SI3).

The broad frequency range of nano-DMA measurements was achieved by combining data obtained performing measurements with three different chirp regimes with different start and end frequencies in order to optimize the sampling frequency for each measurement range and avoid software crashes caused by processing large amounts of data. The frequency range of each chirp was (i) 0.1–10.1 Hz, (ii) 1–1001 Hz, and (iii) 200–20,200 Hz. The sweep time of each chirp was (i) 180 s, (ii) 30 s, and (iii) 1 s. For (i, ii), data were recorded in the time domain with a sampling rate of (i) 1000 Hz and (ii) 10,000 Hz. Data were then transformed in the frequency domain using the fast Fourier transform (FFT). Finally, signals were normalized by the driving signal and smoothed by using the Savitzky-Golay filter. For (iii), the lock-in amplifier accessible in the Cypher ES AFM used was employed. The lock-in sampling rate was set to 5000 Hz, and the low-pass filter time constant was set to 100 Hz. Chirps (i)–(iii) were performed both out of contact with the sample as a reference measurement and in contact with the sample for a sample measurement. At least three reference and sample measurements were performed, and *k*′, *k*″, *E*′, *E*″, and tan δ were calculated using the average amplitude and phase of the reference and sample measurements. The resulting signals from (i) to (iii) were then combined in order to quantify sample viscoelasticity over the entire frequency range.

For sample chirps, the sample was indented with an approach/withdraw velocity of 1 μm s^–1^ and a trigger point of 100 nN. Approach and withdrawal times were 5 s. The withdrawal distance was set to 3 μm. After the 5 s approach, an exponential chirp was applied to the sample. Contact points were identified using the force–indentation variation (FIV) method,^60^ and corrected manually in the event that the FIV method did not accurately identify the contact point. The same procedure was followed for both PT- and PE-AFM nano-DMA measurements.

For PT-AFM nano-DMA experiments, PT actuation was achieved by focusing an excitation laser (EL, blueDrive, Oxford Instruments Asylum Research, 405 nm) at the base of the AFM cantilever (see Supporting Information SI4 for details on positioning the PT-EL). The DC voltage applied to the PT-EL photodiode was set to 4 V, and the AC voltage was set to 1 V for AC160 and 0.1 V for AC240 cantilevers to obtain similar reference amplitudes between cantilevers. Reference measurements were collected approximately 500 μm above the sample surface. See Supporting Information SI4 for details on the effect of reference measurement distance above the sample.

For PE-AFM nano-DMA experiments, PE actuation was achieved by placing an external piezo actuator (PL088.31 PICMA Chip Actuators, Physik Instrumente Ltd., 10 mm × 10 mm × 2 mm) underneath the sample, as in other studies.^18−20,26^ The piezo actuator was secured to a metallic disk with double-sided tape. The SBR sample and a thin glass slide for reference measurements were placed on a metallic disk. The disk containing the sample was fixed with double-sided tape on top of the piezo actuator. The piezo actuator was then connected to the AFM electronics to control piezo actuator motion, with an applied AC voltage of 1 V. Typical resulting cantilever motion can be found in Supporting Information SI1.

Data analysis and visualization for macroscale DMA, as well as PT- and PE-AFM nano-DMA measurements, were performed in Python with home-built codes.

AM–FM AFM imaging was performed on dry SBR samples as a control for PT-AFM nano-DMA measurements. AM–FM AFM creates high-resolution maps of sample viscoelasticity by oscillating a cantilever at two eigenmodes.^15^ Sample tan δ is measured at the lower frequency (*f*_1_) by the first eigenmode.^15,56,57^ Sample *E*′ is measured by both modes, but the higher frequency mode (*f*_2_) contributes most to the contact stiffness, and hence the value of *E*′.^14^ Therefore, *E*′ corresponds to sample properties at the higher frequency (*f*_2_).^14,15,58^ The Hertz contact model is applied in order to measure sample *E*′, but not tan δ.^14,15,56−58^ In these experiments, PT actuation was used to achieve AM–FM AFM’s bimodal cantilever excitation.

For SBR AM–FM AFM, both AC240 (nominal *k*_c_, 2 N/m, *k*_c_, 2–50 N/m, *f*_1_ −70 kHz, *f*_2_ −400 kHz) and AC160 (nominal *k*_c_, 26 N/m, *k*_c_, 2–364 N/m, *f*_1_ −300 kHz, *f*_2_ −1.5 MHz) were used. Cantilevers were excited by using the same PT-EL used for PT-AFM nano-DMA measurements. Multiple spots on the SBR surface were scanned, resulting in a total of 25 AM–FM AFM images taken with AC240 and 13 with AC160. After the experiment, all AM–FM AFM images of SBR topography were flattened using Asylum Research software version 16.10.208 in Igor Pro software version 6.38B01 in order to remove any variations in sample topography that were not due to SBR features. This flattening was done by hand in order to avoid introducing flattening artifacts. The processed files were then analyzed by a custom script in MATLAB R2019b that calculated tan δ and *E*′ via the formulas in refs (14,15,56−57,58) and then compared AM–FM AFM measurements to macroscale DMA control values at the relevant frequency.

## Data Availability

Data are available upon request from the corresponding author.

## Supporting Information Available

SI1: Spurious peaks in PE, but not PT-AFM nano-DMA measurements; SI2: contact models used for *E** calculation; SI3: analysis of chirp signals to excite the AFM cantilever; SI4: analysis of experimental factors that could affect cantilever and sample responses during PT-AFM nano-DMA measurements; SI5: *k** and tan δ error calculation; and SI6: *E** error calculation (PDF)

## Abbreviations

AC: alternating current
AFM: atomic force microscopy
AM–FM: amplitude modulation–frequency modulation
CR: contact resonance
DC: direct current
DMA: dynamic mechanical analysis
EL: excitation laser
FIV: force indentation variation
FTT: fast Fourier transform
JKR: Johnson–Kendall–Roberts
PE: piezoelectric
PT: photothermal
SBR: styrene–butadiene rubber
TTS: time–temperature superposition
