# Supplementary material for: Nanoscale Rheology: Dynamic Mechanical Analysis over a Broad and Continuous Frequency Range Using Photothermal Actuation Atomic Force Microscopy
Source: Macromolecules. 2024 Jan 16;57(3):1118–27. doi: 10.1021/acs.macromol.3c02052 (PMC10867883; doi:10.1021/acs.macromol.3c02052)
Supplement: Supplementary file 1 — ma3c02052_si_001.pdf [file ma3c02052_si_001.pdf]

Supporting Information

Nanoscale rheology: Dynamic Mechanical Analysis over a broad and continuous frequency range using Photothermal Actuation Atomic Force Microscopy

Alba R. Piacenti[a], Casey Adam[a,b], Nicholas Hawkins[b], Ryan Wagner[c], Jacob Seifert[a], Yukinori Taniguchi[d], Roger Proksch[e], Sonia Contera[a]*

[a]Clarendon Laboratory, Department of Physics, University of Oxford, OX1 3PU Oxford, UK

[b]Department of Engineering Science, University of Oxford, OX1 3PJ Oxford, UK

[c]School of Mechanical Engineering, Purdue University, West Lafayette, Indiana, 47907, USA

[d]Asylum Research, Oxford Instruments KK, Tokyo 103-0006, Japan

[e]Asylum Research-An Oxford Instruments Company, Santa Barbara, California 93117, USA

* Corresponding author. E-mail: sonia.antoranzcontera@physics.ox.ac.uk

<u>This document contains the following sections:</u>

SI1: SPURIOUS PEAKS IN PE, BUT NOT PT-AFM NANO-DMA MEASUREMENTS

SI2: CONTACT MODELS USED FOR E* CALCULATION

SI3: ANALYSIS OF CHIRP SIGNALS TO EXCITE THE AFM CANTILEVER

SI4: ANALYSIS OF EXPERIMENTAL FACTORS THAT COULD AFFECT CANTILEVER AND SAMPLE RESPONSES DURING PT-AFM NANO-DMA MEASUREMENTS

SI5: k* AND tanδ ERROR CALCULATION

SI6: E* ERROR CALCULATION

SI1: SPURIOUS PEAKS IN PE, BUT NOT PT-AFM NANO-DMA MEASUREMENTS

Often in PE excitation, spurious resonances arising from the piezo and the piezo/AFM system add noise to AFM measurements[1–3]. Figure S1 shows typical cantilever amplitude and phase signals obtained with PT and PE-AFM nano-DMA, respectively direct and indirect ways to excite the AFM cantilever. At frequencies above ~1 kHz, PE amplitude and phase exhibit multiple local minima and maxima, which are spurious peaks that limit the frequency range over which PE-AFM nano-DMA can be performed. These spurious peaks are not present for PT-AFM nano-DMA.

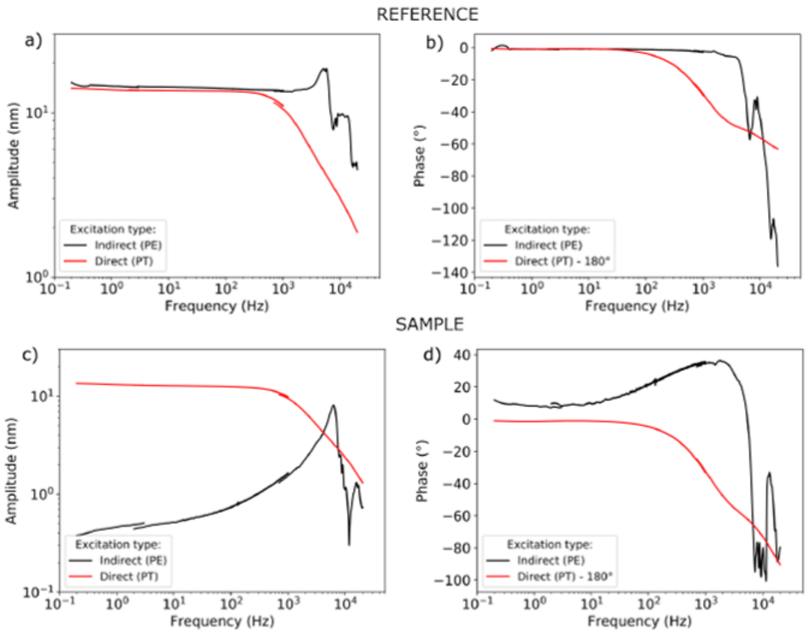

Figure S1. Spurious peaks in PE, but not PT measurements. Cantilever amplitude is shown in (a) for reference measurements and (c) for sample (SBR) measurements. Similarly, cantilever phase is shown in (b) and (d) for reference and sample (SBR) measurements, respectively. PT measurements (red lines) are smooth for all frequencies in the measured range while PE measurements (black lines) exhibit multiple peaks. These spurious peaks hinder mechanical characterization above ~1 kHz using PE actuation.

SI2: CONTACT MODELS USED FOR E* CALCULATION

SIMPLIFICATION OF EQUATIONS FOR HYPERBOLOID INDENTER GEOMETRY

For indenters with hyperboloid shapes, Sun et al.[4] proposed the following model to describe the AFM cantilever-sample contact when adhesion forces are present:

$$d = \frac{aA}{2R_I}\left[\frac{\pi}{2} + arcsin\left(\frac{\left({}^{a}/_{A}\right)^2 - 1}{\left({}^{a}/_{A}\right)^2 + 1}\right)\right] - \sqrt{\frac{2\pi wa}{\tilde{E}}} \quad (S1)$$

$$F = \frac{2\tilde{E}A}{2R_I}\left[aA + \frac{a^2 - A^2}{2}\left(\frac{\pi}{2} + arcsin\left(\frac{\left({}^{a}/_{A}\right)^2 - 1}{\left({}^{a}/_{A}\right)^2 + 1}\right)\right)\right] - \sqrt{8\pi a^3 w\tilde{E}} \quad (S2)$$

Where d is the indentation, F the force, a the contact radius between the indenter (I) and the sample (S), $\tilde{E}$ the reduced Young's modulus, w is the energy of adhesion per unit contact area, and A = R_I cotα, with α being the indenter semi-vertical angle and R_I the indenter (AFM tip) radius.

For the AC160 cantilevers used in this study, $a_1 \approx 200$ nm (details on obtaining a_1 are provided in SI6, which discusses calculating the error in E' and E'') and A ≈ 22 nm (for α = 17.5°, R_I = 7nm). Therefore, $a \gg A$ and the following approximation can be used:

$$arcsin\left(\frac{\left({}^{a}/_{A}\right)^2 - 1}{\left({}^{a}/_{A}\right)^2 + 1}\right) \approx arcsin(1) = \frac{\pi}{2} \qquad (S3)$$

Using this approximation, Eqs. (S1) and (S2) simplify to:

$$d = \frac{aA\pi}{2R_I} - \sqrt{\frac{2\pi wa}{\tilde{E}}} \qquad (S4)$$

$$F = \frac{2\tilde{E}A}{2R_I}\left[aA + \frac{a^2 - A^2}{2}\pi\right] - \sqrt{8\pi a^3 w\tilde{E}} \qquad (S5)$$

"*TWO-POINTS METHOD*" FOR SHERICAL, HYPERBOLOID, AND CONICAL INDENTERS

In this section, equations used for the application of the "*two-points method*"[4] using specific points in force-indentation curves are provided for indenters with different geometries. As explained in the main text (and Figure 4), these specific points are the point of zero load "0", the point around which dynamic oscillations occur "1", and the point of zero deformation "2".

Spherical indenter geometry

For indenters with a JKR spherical contact, the contact radius can be calculated as follows[5]:

$$a^3 = \frac{\tilde{R}}{K}\left(F + 3\pi\tilde{R}w + \sqrt{6\pi w\tilde{R}F + \left(3\pi w\tilde{R}\right)^2}\right) \qquad (S6)$$

With $\tilde{R} = \frac{R_I R_S}{R_I + R_S} \approx R_I$ given that the size of the sample (S) is much larger than that of the indenter (I).

Equation S6 can then be used to calculate the contact area at different points along the force indentation curve in order to apply the "*two-points method*" for the spherical JKR contact as follows[4,5]:

Indentation can be written in terms of a and a_0[4,5]:

$$d = \frac{a^2}{\tilde{R}}\left[1 - \frac{2}{3}\left(\frac{a_0}{a}\right)^{\frac{3}{2}}\right] \tag{S7}$$

$$a_0^3 = \frac{6w\pi\tilde{R}^2}{K} \tag{S8}$$

By using point "2" and point "0", K can be calculated as[4]:

$$K = -\frac{F_2}{2}\frac{3}{\sqrt{d_0\tilde{R}}} \tag{S9}$$

And a_0 can be calculated from Eq.(S7) as follows[4]:

$$a_0 = \sqrt{3d_0\tilde{R}} \tag{S10}$$

w can be then calculated by combining Eq.(S9) and (S10) with Eq.(S8). Finally, a can be calculated with Eq.(S6).

Hyperboloid and conical indenter geometry

Calculated in point "0", Eq.(S4) becomes:

$$\sqrt{\frac{2a_0w\pi}{\tilde{E}}} = \frac{a_0A}{2R_I}\pi - d_0 \tag{S11}$$

From which:

$$\frac{w}{\tilde{E}} = \frac{\left(\frac{a_0A}{2R_I}\pi - d_0\right)^2}{2a_0\pi} \tag{S12}$$

Eq.(S5) calculated in point "0" becomes:

$$F_0 = 0 = \frac{A}{2R_I}\left(a_0 A + \frac{a_0^2 - A^2}{2}\pi\right) - a_0\sqrt{\frac{2\pi a_0 w}{\tilde{E}}} \qquad (S13)$$

Applying equation (S11) to equation (S13) gives the following relation:

$$\frac{A}{2R_I}\left(a_0 A + \frac{a_0^2 - A^2}{2}\pi\right) - a_0\left(\frac{a_0 A}{2R_I}\pi - d_0\right) = 0 \qquad (S14)$$

From which a_0 can be extracted. The SymPy Python library was used in the data analysis to extract a_0. Once a_0 is known, $\frac{w}{\tilde{E}}$ can be calculated from Eq.(S12).

Calculated in point "1", Eq.(S7) becomes:

$$d_1 = \frac{a_1 A}{2R_I}\pi - \sqrt{2a_1\pi}\sqrt{\frac{w}{\tilde{E}}} \qquad (S15)$$

From which a_1 can be obtained. The SymPy Python library was used in the data analysis to extract a_1.

Eq.(S8) calculated in point "1" becomes:

$$F_1 = 2\tilde{E}\left[\frac{A}{2R_I}\left[a_1 A + \frac{a_1^2 - A^2}{2}\pi\right]\right] - \left[a_1\sqrt{2a_1\pi}\sqrt{\frac{w}{\tilde{E}}}\right] \qquad (S16)$$

With algebraic rearrangement, Eq.(S16) becomes:

$$\tilde{E} = \frac{F_1}{2\left[\frac{A}{2R_I}\left[a_1 A + \frac{a_1^2 - A^2}{2}\pi\right]\right] - \left[a_1\sqrt{2a_1\pi}\sqrt{\frac{w}{\tilde{E}}}\right]} \qquad (S17)$$

w can be then calculated from Eq.(S12)

Similarly, the same procedure used for the hyperboloid indenter can be applied for conical indenters using Eqs.(5,6) with $d_{NA} = \frac{\pi}{2} a \cot\alpha$ and $F_{NA} = \frac{\pi}{2} a^2 \tilde{E} \cot\alpha$ [6].

SI3: ANALYSIS OF CHIRP SIGNALS TO EXCITE THE AFM CANTILEVER

Most AFM techniques that measure nanoscale viscoelasticity use single frequency sinusoidal signals to excite the cantilever, thereby quantifying sample viscoelasticity at specific, discrete frequencies[7-18]. However, in order to

measure viscoelasticity over a continuous range of frequencies, the single-frequency excitation sinusoid can be replaced with chirp signals[19–22]. Chirp signals have already been employed for PE-AFM rheology to provide continuous and wide frequency range spectra up to 10 kHz[21,22] or 30 kHz[19].

Fig. S2 shows the amplitude and phase of a typical PT-AFM nano-DMA reference measurement obtained by exciting the cantilever with a linear and an exponential, also called logarithmic, chirp signal. Fringes are present at frequencies less than 100 Hz in the amplitude and phase of cantilevers excited with linear chirp signals. These fringes are not present when exponential chirps are used. For this reason, exponential chirps should be used to excite the cantilever during PT-AFM nano-DMA experiments.

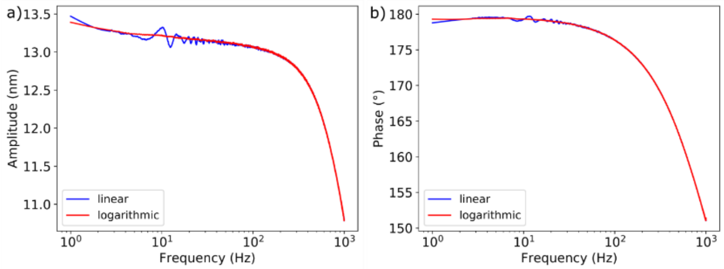

Figure S2. Comparison of cantilever oscillations excited by linear or exponential, also called logarithmic, chirps. The amplitude (a) and phase (b) of cantilever oscillations driven by linear chirp signals (blue line) and logarithmic chirp signals (red line) are shown. Fringes appear in cantilever amplitude and phase at frequencies less than 100 Hz for cantilevers driven by a linear chirp.

SI4: ANALYSIS OF EXPERIMENTAL FACTORS THAT COULD AFFECT CANTILEVER AND SAMPLE RESPONSES DURING PT-AFM NANO-DMA MEASUREMENTS

A detailed analysis of factors that could influence the cantilever and sample responses during a PT-AFM nano-DMA experiment is performed in this section. These factors include (A) PT-EL position on the cantilever, (B) PT-EL spot drift, (C) PT-EL power, (D) PT-EL drive amplitude, (E) effect of the trigger point, the extent of the stimulus applied to the sample, (F) cantilever approach/withdraw velocity, (G) cantilever selection and the relationship between sample vs. cantilever stiffness, and (H) the distance from the sample surface at which the reference measurement is collected.

Finally (I), this analysis is used to recommend a procedure that should be applied to each new sample in order to optimize PT-AFM nano-DMA control parameters and ensure reliable PT-AFM nano-DMA quantification of sample viscoelasticity.

A. Effect of PT Laser Position

PT excitation laser (EL) position on the cantilever alters the amplitude of coated[23–27] and uncoated[24,27,28] cantilever oscillations, and is important in correctly quantifying material stiffness[25]. Therefore, PT-EL positioning could potentially alter PT-AFM nano-DMA measurement values. To investigate whether PT-AFM nano-DMA measurements are robust to different PT-EL positions, PT-AFM nano-DMA was performed on the SBR with a variety of different PT-EL positions, shown in Figure S3. For convenience in the following discussion, each PT-EL position is defined by two coordinates. The first coordinate represents position along the cantilever's length, x/L, where x/L = 1 is at the end of the cantilever and x/L = 0 is at the base. The second coordinate represents position along the cantilever's width, y/W, where y/W = 0 represents the center of the cantilever, and y/W = 1/3 or –1/3 represents the side of the cantilever.

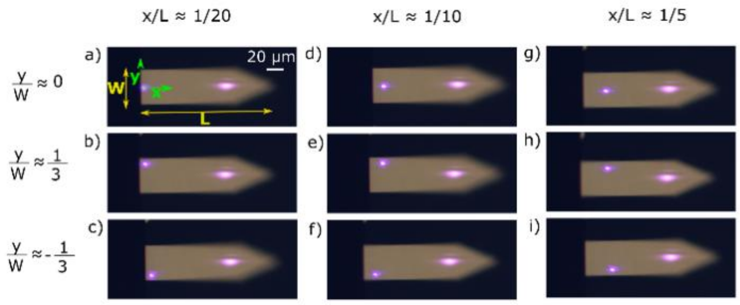

Figure S3. Different PT-EL positions for PT-AFM nano-DMA measurements. The x coordinate, written above the images, represents position along the cantilever's length (L), where x/L = 1 is at the end of the cantilever and x/L = 0 is at the base of the cantilever. Similarly, the y coordinate, written to the left of the images, represents position along the cantilever's width (W), where y/W = ± 1/3 is at the edge of the cantilever, and y/W = 0 is in the center of the cantilever.

Figure S4 shows the effect of PT-EL position on the amplitude and phase signals for both reference and sample measurements. The value of y/W had a negligible effect on cantilever amplitude and phase compared to x/L. At low frequencies, reference phase measurements were robust to PT-EL position. At high frequencies, reference phase

decreased as x/L→ 1. Sample phase measurements followed a similar trend to reference measurements, but the effect was less pronounced. For both reference and sample measurements regardless of frequency, cantilever amplitude increased as x/L → 1.

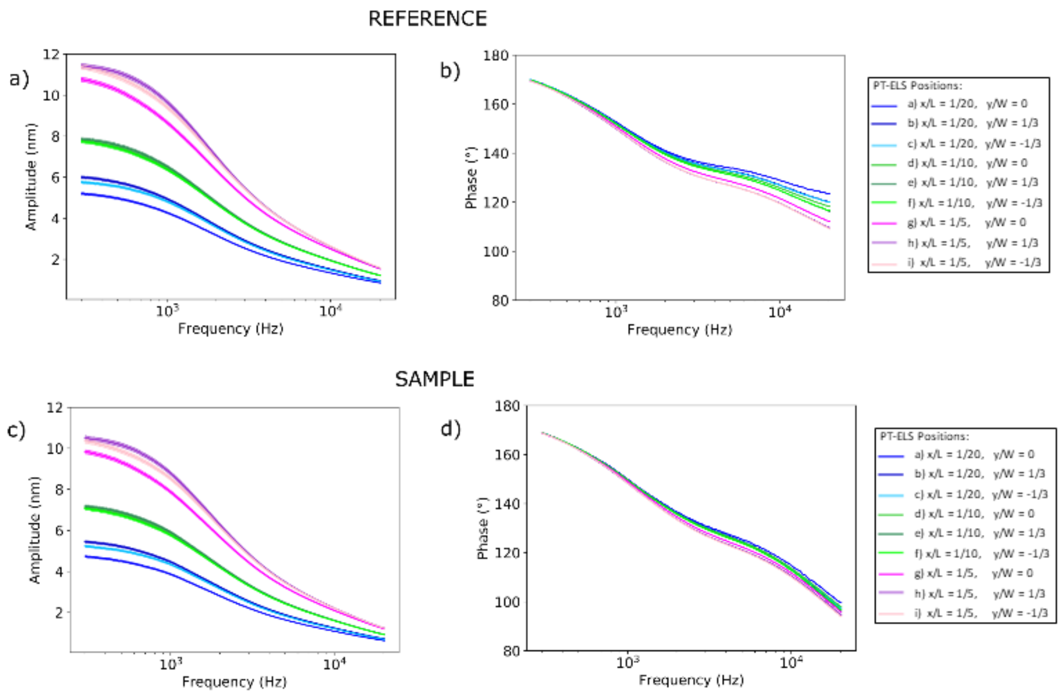

Figure S4. Effect of PT-EL position on cantilever amplitude and phase. Reference amplitude and phase are shown in (a) and (b), respectively. Similarly, sample amplitude and phase are shown in (c) and (d), respectively. PT-EL positions displayed in the legend correspond to those in figure S3.

Figure S5 shows the effect of PT-EL position on PT-AFM nano-DMA quantification of SBR tanδ. As with amplitude and phase measurements (Figure S4), y/W did not alter tanδ values significantly. As x/L -> 1, the measured tanδ decreased compared to measurements obtained with smaller x/L values. However, also shown in Figure S5, PT-AFM nano-DMA quantification of sample tanδ agreed fairly well with the macroscale DMA measurements in spite of tanδ shifts due to PT-EL position changes. Therefore, PT-AFM nano-DMA measurements are robust to PT-EL position, in spite of the differences in cantilever amplitude and phase. However, the combined results of Figures S4 and S5 suggest that PT-EL positioning at x/L ~ 1/10 is ideal because cantilever amplitude is highest in this position and the measured tanδ has the best agreement with the macroscale DMA control. While y/W had little effect on cantilever

motion, x/L ~ 1/10 and y/W ~ 0 is preferable, because this position has already been shown to provide reliable off-resonance mechanical measurements[25]. Therefore, PT-EL positioning at x/L ~ 1/10 and y/W ~ 0 should be used during PT-AFM nano-DMA measurements.

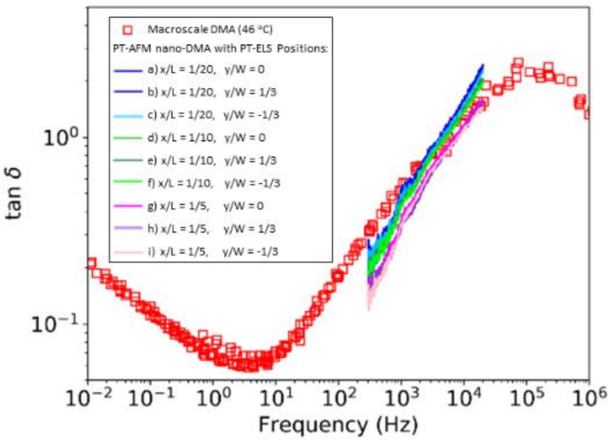

Figure S5. Effect of PT-EL position on loss tangent quantification. SBR loss tangent (tanδ) measurements collected with different PT-EL positions are shown as lines. PT-EL positions displayed in the legend correspond to those in Figure S3. Macroscopic DMA control values are shown as unfilled red squares.

B. Effect of Laser Spot Drift

During an AFM experiment, it is possible that the PT-EL and detection laser positions on the cantilever could drift. Therefore, it is necessary to determine whether PT-AFM nano-DMA measurements are robust to laser spot drift. After zeroing the photodiode, the following procedure was adopted to determine the effects of laser spot drift on PT-AFM nano-DMA measurements. First, a reference measurement was performed 500 µm above the sample surface. Second, the tip was lowered into contact with the sample and a sample measurement was performed. Third, the cantilever was raised to 600 µm above the surface, then lowered to 550 µm, then again lowered to 500 µm above the sample surface. In this third step, the cantilever was not immediately moved to 500 µm above the sample in order to avoid measuring the effects of cantilever backlash, defined as temporary changes in cantilever position due to motor direction reversal, and thereby only measure effects due to laser spot drift. The entire procedure was repeated five times without zeroing the photodiode.

Figures S6 and S7 show the effect of PT-EL spot drift on the amplitude and phase of reference and sample measurements, as well as the relative measurement errors. For a signal x, the relative error is |x – M| / M, where M is the mean of all x. Relative errors were less than 1% and 0.25% for amplitude and phase. Gibbs phenomena occur at the edges of the measured frequency range, resulting in larger relative errors. This larger error at the edges of the frequency range is therefore expected and can be discounted.

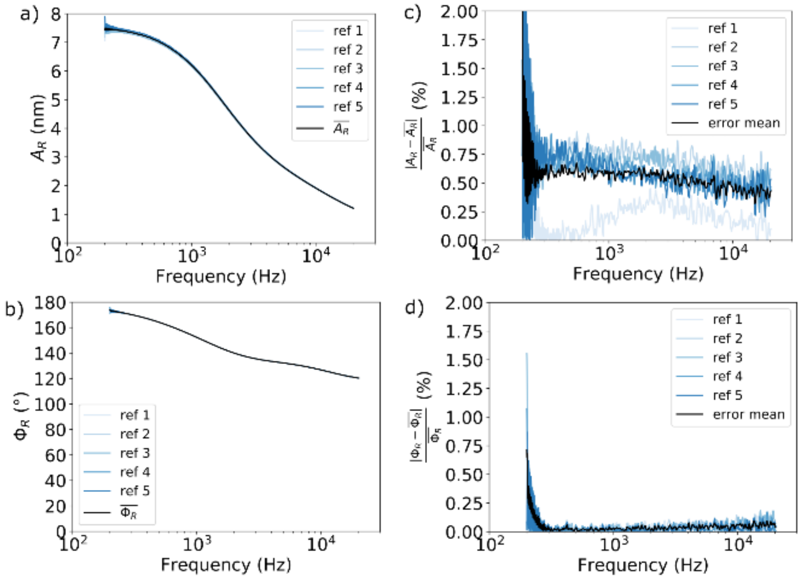

Figure S6. Effect of PT-EL spot drift on reference measurement amplitude (A) and phase (ϕ). Reference (subscript R) A and ϕ measured throughout the course of laser spot drift are shown in (a) and (b), respectively. The relative error between A and ϕ measurements subjected to different drifts are shown in (c) and (d), respectively.

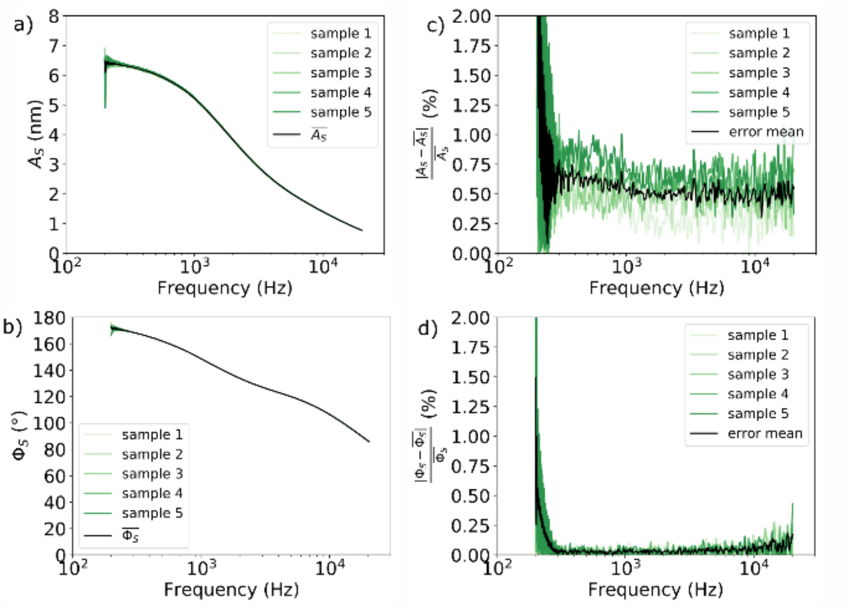

Figure S7. Effect of PT-EL spot drift on sample measurement amplitude (A) and phase (ϕ). Sample (subscript S) A and ϕ measured throughout the course of laser spot drift are shown in (a) and (b), respectively. The relative error between A and ϕ measurements subjected to different drifts are shown in (c) and (d), respectively.

C. Effect of PT Laser Power

The power of the PT-EL affects how much the cantilever bends, and therefore could potentially alter PT-AFM nano-DMA measurements. In order to determine whether PT-EL power affected quantification of sample viscoelasticity, PT laser power was modulated via filters built into the Cypher AFM. Figure S8 shows cantilever amplitude and SBR tanδ measured by PT-AFM nano-DMA performed at 100%, 30%, and 10% of the maximum PT-EL power. For all three powers, a drive amplitude of 1V was used. Reference and sample amplitudes decreased with decreasing PT-EL power. As PT-EL power decreased, measured tanδ values shifted slightly to lower frequencies, and exhibited increased noise. However, regardless of PT-EL power, the overall shape of tanδ vs. frequency remained similar to the macroscopic DMA control. The shift to lower frequencies for lower PT-EL powers is likely due to the fact that lower PT-EL powers heat the sample less than higher PT-EL powers. The increased tanδ noise at lower PT-EL powers is likely due to the lower cantilever amplitudes, and hence worse signal-to-noise ratios at lower PT-EL powers. For measurements at high frequencies collected at 10% PT-EL power, cantilever amplitude decreased below the nm scale. This amplitude decrease is the most likely reason why PT-AFM nano-DMA tanδ results deviate from the DMA

control at high frequencies with 10% PT-EL power. Together, these results suggest that, as long as cantilever amplitude is sufficiently high (nm or higher), PT-AFM nano-DMA is robust to laser power. If PT-EL power is too low, discontinuities and additional peaks appear in the measured tanδ. On the other side, it should be also considered that if PT-EL is too high there is the risk of introducing unwanted non-linear viscoelastic effects due to too large cantilever oscillations (and consequent sample deformations). Therefore, it is important to perform PT-AFM nano-DMA at different PT-EL powers for each new sample, in order to determine the optimal PT-EL power at which to perform the measurements.

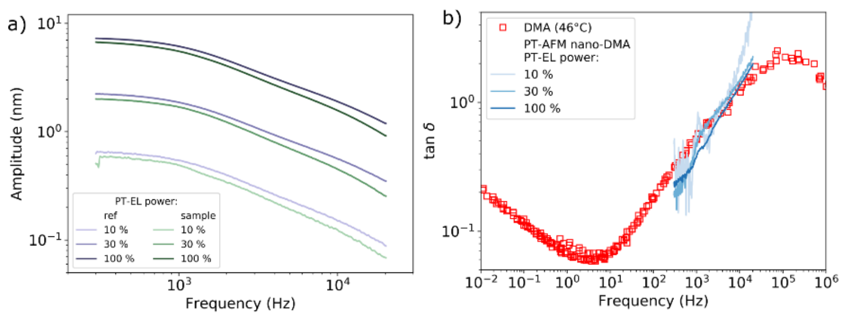

Figure S8. Effect of PT-EL power on PT-AFM nano-DMA measurements. Cantilever amplitude at different PT-EL powers is shown in (a). SBR tanδ measured by PT-AFM nano-DMA at different powers (lines) and macroscopic DMA (unfilled squares) are shown in (b).

D. Effect of Drive Amplitude

As described in the previous section, ensuring that cantilever amplitude is at least in the nm range is critical in correctly quantifying sample viscoelasticity via PT-AFM nano-DMA. Users control cantilever amplitude by changing PT-EL power, as in the previous section, or more commonly by changing the drive amplitude applied to the PT-EL. In order to further investigate the effects of cantilever amplitude on PT-AFM nano DMA measurements, SBR measurements were performed with a drive amplitude of: 0.1V, 0.25V, 0.50V, 1.00V, and 1.5V. At each drive amplitude, two reference and two sample measurements were performed.

Figure S9 shows the relationship between drive amplitude and the resulting reference and sample amplitudes during PT-AFM nano-DMA. Measurement amplitudes were linearly proportional to the drive amplitude (Figure S9(c,d)).

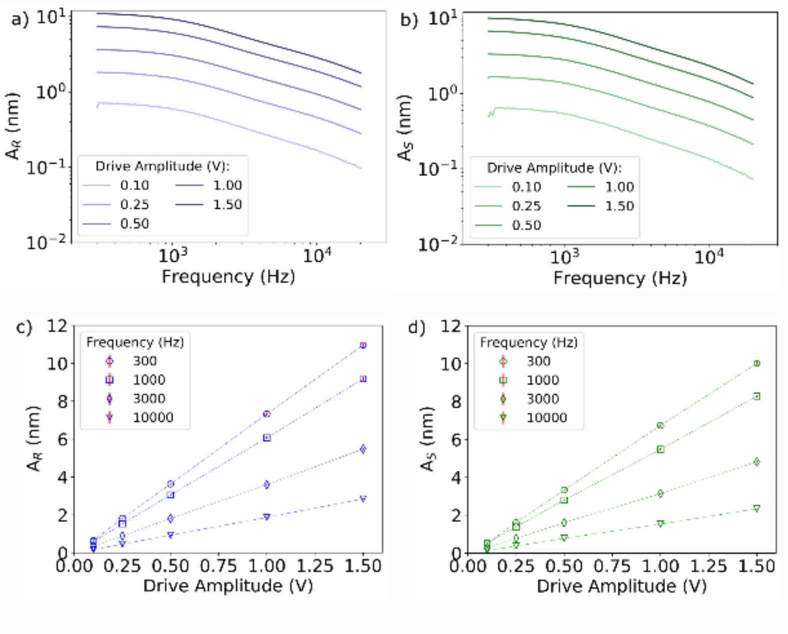

Figure S9. Effect of drive amplitude on cantilever amplitude. The amplitude (A) of cantilever oscillations during reference (R) and sample (S) measurements are shown in (a) and (b), respectively. The relationship between A and drive amplitude at specific frequencies is shown in (c) and (d) for R and S measurements, respectively.

Since cantilever amplitude increased with drive amplitude, it is possible that increasing the drive amplitude too much will result in sample deformations outside of the SBR's linear viscoelastic regime. If PT-AFM nano-DMA measurements occur in the linear viscoelastic regime of a sample, a linear relationship should exist between the reference and sample amplitudes because the response of the sample to the stimulus is linear. That is, the amplitude of the oscillatory force applied to the sample ($\propto A_R$) will cause a linear increase in the oscillatory deformation of the sample (A_S). It is important to note that the slope of this relationship at different frequencies might vary because a sample's viscoelastic response is frequency dependent. As shown in figure S10, measurements were performed in the SBR's linear viscoelastic regime for all drive amplitudes. The slope of the line at different frequencies varied, indicating differences in SBR viscoelasticity at different frequencies.

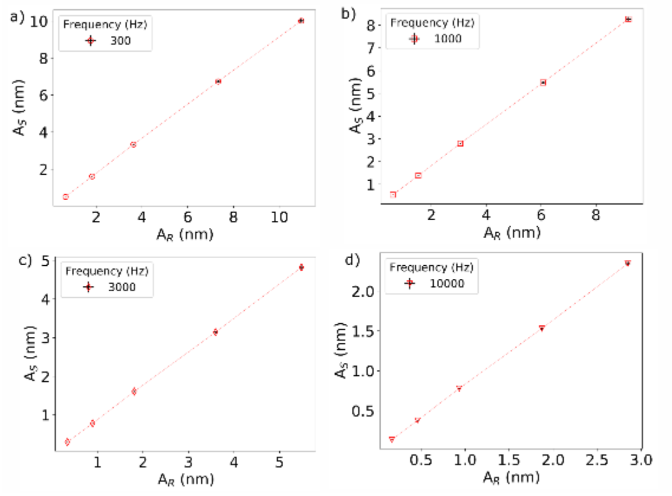

Figure S10. Linear viscoelasticity of the SBR. If PT-AFM nano-DMA is performed in the linear viscoelastic regime of a sample, a linear relationship should exist between cantilever amplitudes (A) during the sample (S) and reference (R) measurements. This line is shown in (a)-(d) at frequencies of 300 Hz, 1,000 Hz, 3,000 Hz, and 10,000 Hz.

SBR tanδ measured at each drive amplitude were similar, as shown in Figure S11, validating that the measurements were performed within the linear viscoelastic regime of the sample. However, as drive amplitude decreased, the resulting tanδ curves grew increasingly noisy. Therefore, for any given sample, if tanδ measurements appear noisy or bumpy, it may be a sign that the measurement amplitude is too low, and that the drive amplitude should therefore be increased.

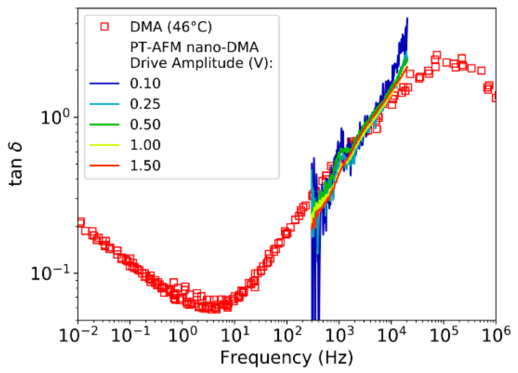

Figure S11. Effect of drive amplitude on loss tangent (tanδ) measurements. SBR tanδ measured by PT-AFM nano-DMA at different drive amplitudes (lines) and macroscopic DMA (unfilled squares) are shown.

E. Effect of Force Trigger Point

As mentioned in the previous section, a sample's response to stimulus depends on whether sample deformations caused by the stimulus fall within the sample's linear viscoelastic regime. Initial sample indentation, before the chirp oscillation is applied, can potentially affect PT-AFM nano-DMA results if the applied force deforms the sample beyond the linear viscoelastic regime. In order to determine the effects of the initial applied force on SBR PT-AFM nano-DMA, sample measurements were performed at different force trigger points of: 10 nN, 25 nN, 50 nN, 75 nN, 100 nN, 250 nN, 500 nN, and 1,000 nN, which led to increasing indentation depths. All sample measurements were compared to the same reference measurement.

As shown in figure S12 (a,b), k‘ and k’’ increased with increasing initial applied force. This observation is unsurprising, since stiffness is an extensive quantity that depends on the geometry of the contact which is affected by the indentation depth. Additionally, k′ at high frequencies exhibits a sudden decrease for large trigger points for this sample and cantilever combination. This change in k’ causes a sudden increase, similar to an asymptote, in tanδ (Figure S12(c)). The appearance of the asymptote-like changes in k’ and tanδ suggest that higher trigger points resulted in sample deformations outside the SBR's linear viscoelastic regime. Therefore, it is important to ensure that the trigger point selected for PT-AFM nano-DMA measurements is within the sample's linear viscoelastic regime.

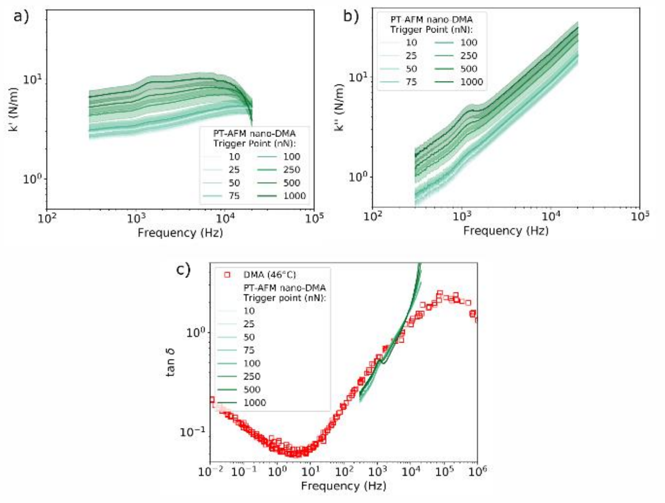

Figure S12. Effect of initial applied force on PT-AFM nano-DMA measurements. SBR k' and k'' measured at different initial applied forces are shown in (a) and (b), respectively. The resulting tanδ is shown in (c). PT-AFM nano-DMA measurements are shown as lines, while macroscopic DMA control measurements are shown as unfilled squares.

F. Effect of Approach and Withdraw Velocity

The velocity at which a sample is indented can affect a sample's viscoelastic response. For slower velocities, polymers within the sample have more time to rearrange in response to the tip. For faster velocities, polymers have less time to rearrange. In order to determine whether cantilever approach/withdraw velocity altered PT-AFM nano-DMA measurements, the SBR sample was indented at velocities of: 0.1 μm s^{-1}, 1.0 μm s^{-1}, 10.0 μm s^{-1}, 50.0 μm s^{-1}, and 100 μm s^{-1}. Approach time was set to 5 s for all velocities except 0.1 μm s^{-1}, where the approach time was reset to 35 s in order to give the cantilever enough time to contact the sample before applying the oscillations. As shown in figure S13, approach/withdraw velocity did not alter PT-AFM nano-DMA measurements in this experiment.

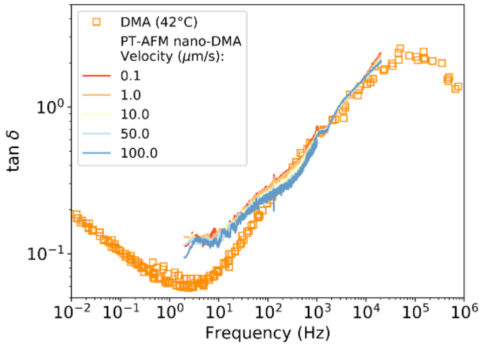

Figure S13. Effect of approach/withdraw velocity on PT-AFM nano-DMA measurements. SBR tanδ measured by PT-AFM nano-DMA with different cantilever approach/withdraw velocities are shown as lines. Macroscopic DMA control values are shown as unfilled squares.

G. Effect of Contact Stiffness

The ratio of dynamic stiffness (k*) and cantilever stiffness (k_c) can affect PT-AFM nano-DMA measurements. If |k*| >> k_c, the sample is not indented enough to measure. If |k*| << k_c, the measurement is insensitive to changes in sample modulus. Additionally, differences in optical lever calibration between the freely vibrating cantilever

(reference) amplitude and the in-contact vibrating cantilever (sample) amplitude must be small for the equations used to calculate PT-AFM nano-DMA k' and k'' (equations 1 and 2) to apply[25]. To minimize these differences in optical lever calibration, the cantilever used should satisfy $k_C > |k^*|$ [25].

To examine the effect of cantilever selection and contact stiffness on SBR measurements, PT-AFM nano-DMA was performed on the SBR using different cantilevers. Three cantilevers were tested. The first cantilever was an AC160TSA-R3 (Olympus, nominal tip radius 7 nm, tetrahedral tip shape, calibrated spring constant 16.93 N m^{-1}, resonance frequency 267 kHz), the second cantilever was an AC240TSA-R3 (Olympus, nominal tip radius 7 nm, tetrahedral tip shape, calibrated spring constant 1.13 N m^{-1}, resonance frequency 55 kHz,), and the third cantilever was a biosphere™-NT_B2000_v0010 (Nanotools, nominal tip radius 2 µm, spherical tip shape, calibrated spring constant 38.92 N m−1, resonance frequency 284 kHz.). Cantilever stiffness was calibrated by indenting a hard substrate, then using the thermal noise method[30]. The drive amplitude was varied for each cantilever so that reference measurements collected by all three cantilevers had approximately the same reference amplitude.

Three reference and three sample measurements were collected for each cantilever and averaged. The resulting k', k'', and tanδ values are shown in figure S14. Biosphere k' and k'' are higher than those of the AC240 and AC160 cantilevers, due to the altered contact geometry of a 2 µm tip (Biosphere) vs. a 7 nm tip (AC160 and AC240). Asymptote-like deviations from control DMA measurements appear for Biosphere and AC240 measurements when $|k^*|$ approaches the stiffness of each cantilever. The same does not occur for AC160 cantilevers because $|k^*|$ was always lower than AC160 cantilever stiffness. Therefore, these results demonstrate that, as long as $k_C > |k^*|$ over the entire frequency range, PT-AFM nano-DMA can accurately quantify sample viscoelasticity. For each sample, it is therefore important to compare $|k^*|$ to cantilever stiffness in order to ensure this condition holds.

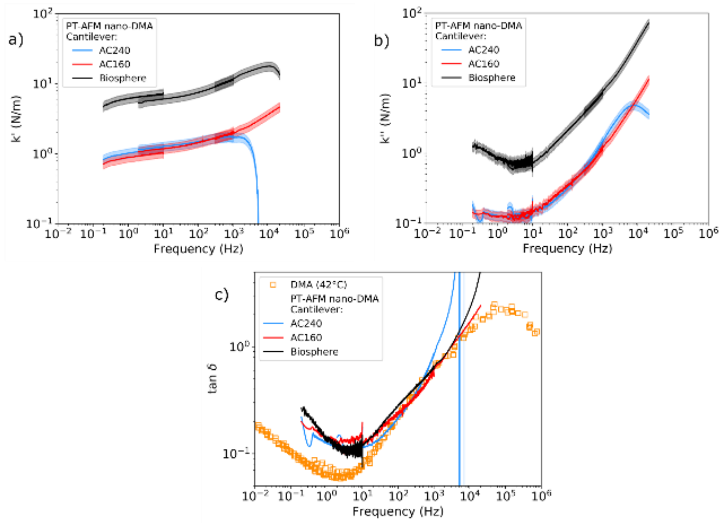

Figure S14. Effect of cantilever selection and contact stiffness on PT-AFM nano-DMA measurements. PT-AFM nano-DMA measurements performed with different cantilevers, and therefore different contact stiffness, are shown as lines. The resulting k', k'', and tanδ are shown in (a-c), respectively. Macroscale DMA control measurements are shown as unfilled squares.

H. Effect of Reference Measurement Height

Surface effects, such as the moisture layer over dry samples or squeeze film damping, might influence reference measurements if the reference is collected too close to a sample's surface. In order to determine the effect of reference measurement height, three reference measurements were collected and averaged at a distance of 500 µm, 400 µm, 300 µm, 200 µm, 100 µm, 50 µm, 3 µm, and 1 µm from the sample surface. After collecting four reference measurements at each height, four sample measurements were performed and averaged. The average sample measurement was then compared to the average reference measurement at each height.

Figure S15 shows the amplitude and phase of each reference measurement. All reference measurement amplitudes, regardless of reference height, were within the calculated error (see section SI4 B) of one another. Therefore, reference height did not affect the reference amplitude. However, as shown in Figure S15(b), references collected 1 µm and 3 µm from the sample surface had an approximate phase difference of 0.2° from the other reference measurements. This observation suggests that the SBR sample may have influenced the 1 µm and 3 µm reference measurements.

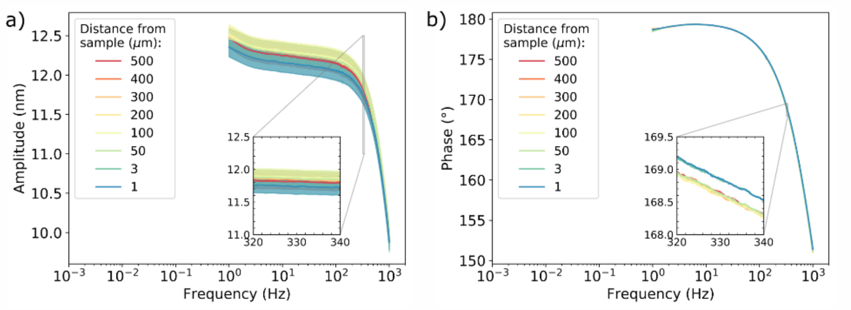

Figure S15. Effect of reference measurement height on cantilever amplitude and phase. Cantilever amplitude and phase are shown in (a) and (b), respectively, for measurements collected at different heights above the sample surface.

Figure S16 shows the effect of reference height on PT-AFM nano-DMA quantification of SBR viscoelasticity. Quantification of SBR tanδ agrees with the DMA control measurements for all reference heights, save for references collected 1 μm and 3 μm from the sample surface. This observation supports the notion that the closer reference measurements were subject to surface effects. Therefore, PT-AFM nano-DMA measurements of sample viscoelasticity are robust over a wide range of reference heights as long as the reference is not subject to surface layer effects.

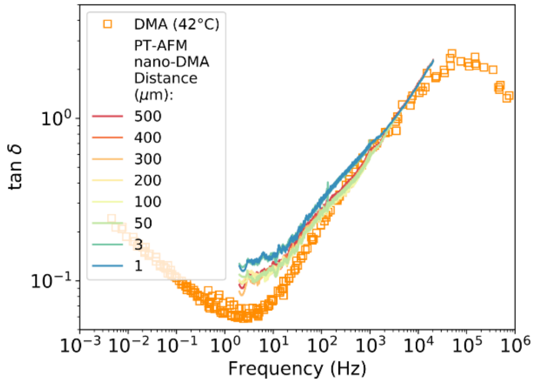

Figure S16. Effect of reference measurement height on the measured tanδ. PT-AFM nano-DMA measurements with references at different heights are shown as lines. Macroscale DMA control measurements are shown as unfilled squares.

I. Summary

In summary, PT-AFM nano-DMA is robust to a variety of factors that can influence the cantilever and sample response. Based on the analyses in this section, when performing PT-AFM nano-DMA on dry samples, it is important

to ensure that the PT-EL is positioned in the center of the cantilever, and roughly 1/10 of the way down the length of the cantilever. It is also important to ensure that (i) cantilever amplitude is large enough to be above the detection noise floor but not so large that sample deformations are beyond the sample's linear viscoelastic regime, (ii) that the initial applied stimulus is in the sample's linear viscoelastic regime, (iii) that |k*| never exceeds cantilever stiffness, and (iv) that the reference is far enough from the sample surface to avoid any surface effects. Incorrect selection of any of the parameters in (i)-(iv) results in incorrect measurement of sample viscoelasticity, including noisy data and asymptotes that appear in the measured k', k'', and tanδ curves. Therefore, when PT-AFM nano-DMA is first performed on a sample, it is ideal to account for these factors by collecting measurements with: different trigger points, different PT-EL powers and drive amplitudes, and different reference heights to find the optimal combination to measure the given sample's viscoelasticity. From these combinations, ideal PT-AFM nano-DMA parameters for the given sample can be selected by minimizing the amount of noise in the measurement (for example tanδ) curves and excluding parameters that result in asymptote-like changes in the curves.

SI5: k* AND tanδ ERROR CALCULATION

The uncertainty dy of a quantity y that is a product or ratio of N different x_i that each have an associated uncertainty dx_i (i = 1, … , $N \in \mathbb{N}$) is calculated as follows[29]:

$$\frac{dy}{|y|} = \sqrt{\sum_{i=1}^{N}\left(\frac{dx_i}{x_i}\right)^2} \qquad (S18)$$

For the PT-AFM nano-DMA components of k* calculated using Eqs. (1,2), the error could arise from uncertainty in cantilever stiffness k_C, and the cantilever amplitude and phase. Since the relative error was much larger for amplitude measurements than for phase measurements (see section SI4 B), only amplitude effects were used to calculate the uncertainty in PT-AFM nano-DMA measurements due to PT-EL spot drift. Therefore, the error in k' and k'' calculated with Eq. (1,2) can be calculated as follows:

$$\frac{dk^j}{|k^j|} = \sqrt{\left(\frac{dk_c}{k_c}\right)^2 + \left(\frac{d\overline{A}}{\overline{A}}\right)^2} \qquad (S19)$$

Here, k^j represents k' and k''. The value of $dk_c \; / \; k_c$ for cantilever's stiffness evaluated with the thermal noise method[30] can be estimated as 0.15[31]. Since the relative error in the amplitude is 0.01, the overall error in PT-AFM nano-DMA amplitude measurements due to laser spot drift is 1.4%, calculated as follows:

$$\frac{d\overline{A}}{\overline{A}} = \sqrt{\left(\frac{dA_R}{A_R}\right)^2 + \left(\frac{dA_S}{A_S}\right)^2} = \sqrt{0.01^2 + 0.01^2} = 0.014 \qquad (S20)$$

Here, R and S indicate a reference or sample measurement, respectively. Therefore, the amplitude error (1.4%) is much smaller compared to the uncertainty in cantilever stiffness (15%).

Recalling that tanδ = k''/k', the error in tanδ can be calculated as follows.

$$\frac{dtan\delta}{tan\delta} = \frac{\partial\left(\frac{\overline{A}sin\Phi}{\overline{A}cos\Phi - 1}\right)}{\partial\overline{A}} d\overline{A} = \frac{tan\delta}{\overline{A}cos\Phi - 1}\frac{d\overline{A}}{\overline{A}} \qquad (S21)$$

SI6: E* ERROR CALCULATION

The error in AFM nano-DMA quantification of sample E' and E'', can be calculated from Eq.(4) as with Eq.(S.18):

$$\frac{dE^j}{E^j} = \sqrt{\left(\frac{dk}{k^j}\right)^2 + \left(\frac{da_1}{a_1}\right)^2} \qquad (S22)$$

where j indicates the storage (') and the loss ('') moduli.

The uncertainty in $a_1(da_1)$ can be written as the contribution of 2 factors: da^{curve}, the error in the quantities used to calculate a_1(i.e. variation of F_1 and d_1), and da^{tip}, the error in the indenter's geometrical parameters (e.g. tip radius, α):

$$da = da^{curve} + da^{tip} \qquad (S23)$$

da_1^{curve} can be calculated as the standard deviation of a_1 estimated over several curves.

da_1^{tip} can be estimated by calculating how much a_1 changes by altering the tip's geometrical parameters compared to the nominal values.

In Figure S17, a_1 values calculated using different tip geometrical parameters for an AC160 cantilever are plotted with their standard deviation obtained from several curves (da_1^{curve}).

Figure S17(a) shows a_1 calculated for an AC160 with the JKR model with indenter radii close to the nominal tip radius (R=7nm). For a blunted cantilever with R=14 nm $da_1^{tip} = |a_1^{R=7nm} - a_1^{R=14}|$. From Figure S17(a), it can be seen that $da_1^{tip}/a_1 \approx 40\% \gg da_1^{curve}/a_1$. Therefore, a 40% error is assumed when calculating E' and E'' via adhesive contact models with spherical indenters.

Figure S17(b) shows a_1 calculated for an AC160 with the hyperboloid model with adhesion (see section SI2) for radii close to the nominal tip radius (R=7nm) and nominal semi-vertical angle (α=17.5°, taken as the half of the tip nominal back angle). The larger contribution to the error is given by the change in α and leads to a relative error of $da_1^{tip}/a_1 \approx 30\% \gg da_1^{curve}/a_1$. Therefore, a 30% error is assumed when calculating E' and E'' via adhesive contact models with hyperboloid and conical indenters.

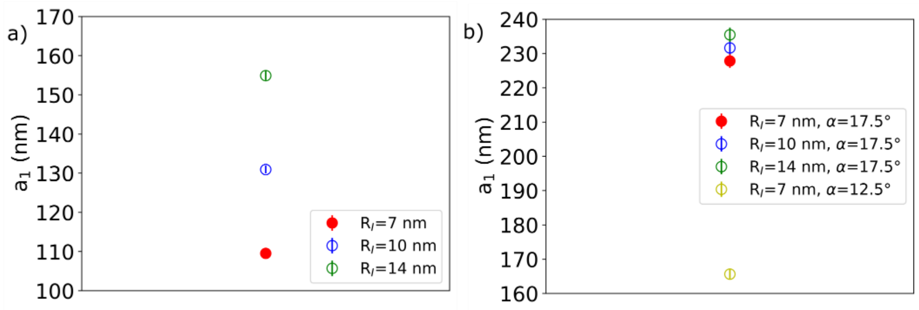

Figure S17. AC160 cantilever contact radius calculated in point 1 (a_1) for different contact models and tip sizes: (a) the JKR model with different tip radii, and (b) the adhesive hyperboloid model with different tip radii and semi-vertical angles.

(1) Schäffer, T. E.; Cleveland, J. P.; Ohnesorge, F.; Walters, D. A.; Hansma, P. K. Studies of Vibrating Atomic Force Microscope Cantilevers in Liquid. *J. Appl. Phys.* **1996**, *80* (7), 3622–3627. https://doi.org/10.1063/1.363308.

(2) Rabe, U.; Hirsekorn, S.; Reinstädtler, M.; Sulzbach, T.; Lehrer, C.; Arnold, W. Influence of the Cantilever

Holder on the Vibrations of AFM Cantilevers. *Nanotechnology* **2006**, *18* (4), 44008.

(3) Labuda, leksander; Cleveland, J.; Geisse, N. A.; Kocun, M.; Ohler, B.; Proksch, R.; Viani, M. B.; Walters, D. Photothermal Excitation for Improved Cantilever Drive Performance in Tapping Mode Atomic Force Microscopy. *Microsc. Anal.* **2014**, *28* (3), 21–25.

(4) Sun, Y.; Akhremitchev, B.; Walker, G. C. Using the Adhesive Interaction between Atomic Force Microscopy Tips and Polymer Surfaces to Measure the Elastic Modulus of Compliant Samples. *Langmuir* **2004**, *20* (14), 5837–5845. https://doi.org/10.1021/la036461q.

(5) Johnson, K. L.; Kendall, K.; Roberts, aAD. Surface Energy and the Contact of Elastic Solids. *Proc. R. Soc. London. A. Math. Phys. Sci.* **1971**, *324* (1558), 301–313.

(6) Sneddon, I. N. The Relation between Load and Penetration in the Axisymmetric Boussinesq Problem for a Punch of Arbitrary Profile. *Int. J. Eng. Sci.* **1965**, *3* (1), 47–57.

(7) Kolluru, P. V; Eaton, M. D.; Collinson, D. W.; Cheng, X.; Delgado, D. E.; Shull, K. R.; Brinson, L. C. AFM-Based Dynamic Scanning Indentation (DSI) Method for Fast, High-Resolution Spatial Mapping of Local Viscoelastic Properties in Soft Materials. *Macromolecules* **2018**, *51* (21), 8964–8978. https://doi.org/10.1021/acs.macromol.8b01426.

(8) Pittenger, B.; Osechinskiy, S.; Yablon, D.; Mueller, T. Nanoscale DMA with the Atomic Force Microscope: A New Method for Measuring Viscoelastic Properties of Nanostructured Polymer Materials. *JOM* **2019**, *71* (10), 3390–3398.

(9) Arai, M.; Ueda, E.; Liang, X.; Ito, M.; Kang, S.; Nakajima, K. Viscoelastic Maps Obtained by Nanorheological Atomic Force Microscopy with Two Different Driving Systems. *Jpn. J. Appl. Phys.* **2018**, *57* (8S1), 08NB08. https://doi.org/10.7567/jjap.57.08nb08.

(10) Ueda, E.; Liang, X.; Ito, M.; Nakajima, K. Dynamic Moduli Mapping of Silica-Filled Styrene–Butadiene Rubber Vulcanizate by Nanorheological Atomic Force Microscopy. *Macromolecules* **2019**, *52* (1), 311–319. https://doi.org/10.1021/acs.macromol.8b02258.

(11) Mahaffy, R. E.; Shih, C. K.; MacKintosh, F. C.; Käs, J. Scanning Probe-Based Frequency-Dependent

Microrheology of Polymer Gels and Biological Cells. *Phys. Rev. Lett.* **2000**, *85* (4), 880.

(12) Alcaraz, J.; Buscemi, L.; Grabulosa, M.; Trepat, X.; Fabry, B.; Farré, R.; Navajas, D. Microrheology of Human Lung Epithelial Cells Measured by Atomic Force Microscopy. *Biophys. J.* **2003**, *84* (3), 2071–2079.

(13) Mahaffy, R. E.; Park, S.; Gerde, E.; Käs, J.; Shih, C. K. Quantitative Analysis of the Viscoelastic Properties of Thin Regions of Fibroblasts Using Atomic Force Microscopy. *Biophys. J.* **2004**, *86* (3), 1777–1793.

(14) Rigato, A.; Miyagi, A.; Scheuring, S.; Rico, F. High-Frequency Microrheology Reveals Cytoskeleton Dynamics in Living Cells. *Nat. Phys.* **2017**, *13* (8), 771–775.

(15) Lherbette, M.; Santos, Á.; Hari-Gupta, Y.; Fili, N.; Toseland, C. P.; Schaap, I. A. T. Atomic Force Microscopy Micro-Rheology Reveals Large Structural Inhomogeneities in Single Cell-Nuclei. *Sci. Rep.* **2017**, *7* (1), 8116.

(16) Nalam, P. C.; Gosvami, N. N.; Caporizzo, M. A.; Composto, R. J.; Carpick, R. W. Nano-Rheology of Hydrogels Using Direct Drive Force Modulation Atomic Force Microscopy. *Soft Matter* **2015**, *11* (41), 8165–8178. https://doi.org/10.1039/c5sm01143d.

(17) Igarashi, T.; Fujinami, S.; Nishi, T.; Asao, N.; Nakajima, and K. Nanorheological Mapping of Rubbers by Atomic Force Microscopy. *Macromolecules* **2013**, *46* (5), 1916–1922. https://doi.org/10.1021/ma302616a.

(18) Nguyen, H. K.; Ito, M.; Fujinami, S.; Nakajima, K. Viscoelasticity of Inhomogeneous Polymers Characterized by Loss Tangent Measurements Using Atomic Force Microscopy. *Macromolecules* **2014**, *47* (22), 7971–7977.

(19) Schächtele, M.; Hänel, E.; Schäffer, T. E. Resonance Compensating Chirp Mode for Mapping the Rheology of Live Cells by High-Speed Atomic Force Microscopy. *Appl. Phys. Lett.* **2018**, *113* (9). https://doi.org/10.1063/1.5039911.

(20) Nia, H. T.; Han, L.; Li, Y.; Ortiz, C.; Grodzinsky, A. Poroelasticity of Cartilage at the Nanoscale. *Biophys. J.* **2011**, *101* (9), 2304–2313.

(21) Nia, H. T.; Bozchalooi, I. S.; Li, Y.; Han, L.; Hung, H.-H.; Frank, E.; Youcef-Toumi, K.; Ortiz, C.; Grodzinsky, A. High-Bandwidth AFM-Based Rheology Reveals That Cartilage Is Most Sensitive to High Loading Rates at Early Stages of Impairment. *Biophys. J.* **2013**, *104* (7), 1529–1537.

(22) Han, B.; Nia, H. T.; Wang, C.; Chandrasekaran, P.; Li, Q.; Chery, D. R.; Li, H.; Grodzinsky, A. J.; Han, L. AFM-Nanomechanical Test: An Interdisciplinary Tool That Links the Understanding of Cartilage and Meniscus Biomechanics, Osteoarthritis Degeneration, and Tissue Engineering. *ACS Biomater. Sci. Eng.* **2017**, *3* (9), 2033–2049.

(23) Ramos, D.; Tamayo, J.; Mertens, J.; Calleja, M. Photothermal Excitation of Microcantilevers in Liquids. *J. Appl. Phys.* **2006**, *99* (12), 124904. https://doi.org/10.1063/1.2205409.

(24) Kiracofe, D.; Kobayashi, K.; Labuda, A.; Raman, A.; Yamada, H. High Efficiency Laser Photothermal Excitation of Microcantilever Vibrations in Air and Liquids. *Rev. Sci. Instrum.* **2011**, *82* (1), 13702. https://doi.org/10.1063/1.3518965.

(25) Wagner, R.; Killgore, J. P. Photothermally Excited Force Modulation Microscopy for Broadband Nanomechanical Property Measurements. *Appl. Phys. Lett.* **2015**, *107* (20), 203111.

(26) Pini, V.; Tiribilli, B.; Gambi, C. M. C.; Vassalli, M. Dynamical Characterization of Vibrating AFM Cantilevers Forced by Photothermal Excitation. *Phys. Rev. B* **2010**, *81* (5), 54302.

(27) Vassalli, M.; Pini, V.; Tiribilli, B. Role of the Driving Laser Position on Atomic Force Microscopy Cantilevers Excited by Photothermal and Radiation Pressure Effects. *Appl. Phys. Lett.* **2010**, *97* (14), 143105. https://doi.org/10.1063/1.3497074.

(28) Nishida, S.; Kobayashi, D.; Kawakatsu, H.; Nishimori, Y. Photothermal Excitation of a Single-Crystalline Silicon Cantilever for Higher Vibration Modes in Liquid. *J. Vac. Sci. Technol. B Microelectron. Nanom. Struct. Process. Meas. Phenom.* **2009**, *27* (2), 964–968. https://doi.org/10.1116/1.3077487.

(29) Farrance, I.; Frenkel, R. Uncertainty of Measurement: A Review of the Rules for Calculating Uncertainty Components through Functional Relationships. *Clin. Biochem. Rev.* **2012**, *33* (2), 49.

(30) Hutter, J. L.; Bechhoefer, J. Calibration of Atomic-force Microscope Tips. *Rev. Sci. Instrum.* **1993**, *64* (7),

1868–1873.

(31) Clifford, C. A.; Seah, M. P. The Determination of Atomic Force Microscope Cantilever Spring Constants via Dimensional Methods for Nanomechanical Analysis. *Nanotechnology* **2005**, *16* (9), 1666.
